# An Evolutionary Arms Race Between *Burkholderia pseudomallei* and Host Immune System: What Do We Know?

**DOI:** 10.3389/fmicb.2020.612568

**Published:** 2021-01-21

**Authors:** Chalita Chomkatekaew, Phumrapee Boonklang, Apiwat Sangphukieo, Claire Chewapreecha

**Affiliations:** ^1^Mahidol-Oxford Tropical Medicine Research Unit (MORU), Bangkok, Thailand; ^2^Bioinformatics and Systems Biology Program, School of Bioresource and Technology, King Mongkut’s University of Technology Thonburi, Bangkok, Thailand; ^3^Wellcome Sanger Institute, Hinxton, United Kingdom

**Keywords:** melioidosis, evolution, burkholderia, host immune system, genetic variants

## Abstract

A better understanding of co-evolution between pathogens and hosts holds promise for better prevention and control strategies. This review will explore the interactions between *Burkholderia pseudomallei*, an environmental and opportunistic pathogen, and the human host immune system. *B. pseudomallei* causes “Melioidosis,” a rapidly fatal tropical infectious disease predicted to affect 165,000 cases annually worldwide, of which 89,000 are fatal. Genetic heterogeneities were reported in both *B. pseudomallei* and human host population, some of which may, at least in part, contribute to inter-individual differences in disease susceptibility. Here, we review (i) a multi-host—pathogen characteristic of the interaction; (ii) selection pressures acting on *B. pseudomallei* and human genomes with the former being driven by bacterial adaptation across ranges of ecological niches while the latter are driven by human encounter of broad ranges of pathogens; (iii) the mechanisms that generate genetic diversity in bacterial and host population particularly in sequences encoding proteins functioning in host—pathogen interaction; (iv) reported genetic and structural variations of proteins or molecules observed in *B. pseudomallei*—human host interactions and their implications in infection outcomes. Together, these predict bacterial and host evolutionary trajectory which continues to generate genetic diversity in bacterium and operates host immune selection at the molecular level.

## Introduction

Melioidosis is a serious and often fatal neglected tropical infectious disease caused by *Burkholderia pseudomallei*, an intracellular bacterial pathogen and also ubiquitous in the environment (Holden et al., 2004). Human hosts can acquire the bacterium after direct environmental exposure either through dermal puncture, ingestion of contaminated food or water supplies, or inhalation of contaminated soil or water aerosols. Following an acquisition, *B. pseudomallei* can replicate in host non-phagocytic and phagocytic cells as well as spreading intracellularly (Willcocks et al., 2016). The bacterium can either kill the human host rapidly (acute melioidosis) or hide within the host body for a long period (chronic melioidosis). With rare exceptions (Abbink et al., 2001; Ralph et al., 2004; Aziz et al., 2020), *B. pseudomallei* is not known to transmit from person to person; indicating that *B. pseudomallei* has not evolved virulence mechanisms through consecutive passage in human hosts. Moreover, *B. pseudomallei* within-host evolution observed in chronic infections are often linked to attenuated virulence (Price et al., 2013; Viberg et al., 2017; Pearson et al., 2020), which is possibly mediated by host immune evasion. While environment exposure can lead to melioidosis, most patients are elderly or have one or more underlying health risks. The most common health condition is diabetes mellitus which presents in up to half of cases (Kronsteiner et al., 2019). Therefore, it is likely that *B. pseudomallei* has acquired virulence genes for human infection before being exposed to the human hosts, and that these virulence factors are most effective when the host has underlying health issues. It is possible that a successful human infection is mediated by bacterial redundant virulence mechanisms that were acquired and maintained in the environment. In this review, we will leverage recent pieces of genomic studies to better understand the evolution of virulence mechanisms in *B. pseudomallei* and host defense system.

## A Multi-Host—Pathogen Interaction

Interactions between free-living amoebae and environmental bacteria have been proposed to provide an evolutionary training platform for intracellular pathogen such as *Legionella pnemophila* (Park et al., 2020), *Listeria monocytogenes* (Schuppler, 2014), and *B. pseudomallei* (Inglis et al., 2000, 2003; Noinarin et al., 2016). Many free-living amoebae including the genera *Acanthamoeba*, *Dictyostelium*, *Naegleria*, and *Paravahlkampfia* share natural habitats with *Burkholderia* bacteria, including *B. pseudomallei* (Inglis et al., 2000, 2003; Noinarin et al., 2016). Amoeba-*Burkholderia* interactions can range from predator-prey to mutualistic relationships. The fate of these interactions is subjected to the species of amoebae host and *Burkholderia* (genotypes), stages of host, as well as external factors that modify the outcomes. The social amoebae *Dictyostelium discoideum* life cycle ranges from unicellular amoebae to multicellular slugs where ten of thousands of single-celled *D. discoideum* aggregate to form a fruiting body. *D. discoideum* can directly ingest *B. pseudomallei* and other *Burkholderia* bacteria as their food; thereby requiring the bacterium to resist host cell phagocytosis, persist inside a unicellular host, and migrate between host cells through the amoeba cytoskeleton. These processes share many similarities with mammalian host infection and likely prime the bacterium for an intracellular lifestyle. The sentinel cells in *D. discoideum*, which make up approximately 1% of slug cells, can use antimicrobial defense systems similar to those employed by phagocytes in the human innate immune system (Zhang et al., 2016). The sentinel cells are capable of releasing reactive oxygen species to lyse the soil bacterium. Moreover, they can also release an extracellular trap—the reticulated nets of DNA carrying antimicrobial granules—to kill the invading bacteria. The extracellular trap is an ancient host-defense mechanism common among phagocytic cells across vertebrates and invertebrates, thereby providing a training ground for *B. pseudomallei* infection in mammalian hosts. External factors such as nutrient availability were shown to determine the fate of *D. discoideum*—*Burkholderia* relationship. The association is beneficial to both parties when the nutrient is scarce. Under nutrient limited conditions, *Burkholderia*—associated *D. discoideum* produced more spores (Brock et al., 2013; DiSalvo et al., 2015) and had an increased uptake of secondary bacteria that can be used as food (Khojandi et al., 2019) which led to a better survival rate than *D. discoideum* without *Burkholderia* association. Under this condition, *Burkholderia* could be detected inside *D. discoideum* spores, which enable the bacterium to better disseminate. The mechanism underlining the decision to kill or cooperate is unclear.

The species of *Burkholderia* and genetic variations within the species have been shown to impact the outcomes of amoeba internalization (Haselkorn et al., 2019). It is possible that genetic variations in *B. pseudomallei* could influence the outcomes of human melioidosis. Heritability scores (Finucane et al., 2015; Hou et al., 2019; Speed et al., 2020) can be used to quantify the proportion of variations in the infection outcomes that can be explained by genetic variations in pathogens and hosts. The technique has been successfully applied to different pathogens (Lees et al., 2017) and human infectious diseases. However, the concept has not been widely adopted for *B. pseudomallei* – host infection. For *B. pseudomallei* infection in human hosts with no comorbidity, we estimated that 8% of host mortality could be explained by *B. pseudomallei* genotypes (*h*^2^ = 0.081, *SE* = 0.050, *p* = 0.018; see Supplementary Text for method). This new result highlights a moderate proportion of infection outcomes being explained by the bacterial genetics, and suggests that a substantial proportion of the outcomes could be explained by host genetics.

Interactions between pathogens and *Homo sapiens* have shaped the evolution of modern humans (Karlsson et al., 2014). Currently, less is known about how melioidosis has shaped the human populations, particularly in melioidosis endemic areas. The host immunity could function as a general defense against all invading pathogen mediated by the innate immune system, or a pathogen-specific defense facilitated through an adaptive immune response. For innate immune response, it is possible that interactions between human hosts and other common pathogens may have driven selection on host defense pathways that affect resistance. These may include human interactions with parasites or bacteria that cause malaria (Shimizu et al., 2000; Malaria Genomic Epidemiology Network, 2008; Timmann et al., 2012; Kariuki et al., 2020), tuberculosis (Mahasirimongkol et al., 2012; Thye et al., 2012), or sepsis (Southeast Asia Infectious Disease Clinical Research Network, 2017; Sweeney et al., 2018) in many tropical and subtropical countries, and cholangiocarcinoma (Zou et al., 2014) in Southeast Asia. Using *h*^2^, previous works estimate that genetic variations in human host can explain between 32 and 52% of infectious disease susceptibilities, depending on the population studied and the infectious agents (Cooke and Hill, 2001; Jia et al., 2019). Candidate gene approaches have determined host risk- and protective genetic markers for melioidosis infection (West et al., 2012, 2013; Myers et al., 2014; Chaichana et al., 2017; Dickey et al., 2019), many of which were previously identified as gene targets for other common pathogen infections. To cover melioidosis-specific, as well as broad-pathogen host factors, systemic and reproducible genome-wide scans are yet to be conducted.

## Natural Selection in Genes Required for Host-Pathogen Interaction

Given an increasing availability of *B. pseudomallei* and host genomes, a genome-wide scan for signatures of selection, their directions (purifying, balancing, and positive selection) and their magnitudes could be very useful to investigate host-pathogen protein-protein interactions. As both host and *B. pseudomallei* migrate, detection of directional selection can be challenging after a population bottleneck. A bottleneck leads to reduced levels of genetic variation and a subsequent loss of a selection signal. For a soil microbe like *B. pseudomallei*, bacterial migration was shown to be infrequent, with major movements tracked and dated (Pearson et al., 2009; Price et al., 2016; Chewapreecha et al., 2017). The bacterial genome evolution is correlated with a strong geographical signal, with the highest diversity observed in Australia and reduced diversity following dissemination out of the Australian origin (Chewapreecha et al., 2017). Nevertheless, frequent recombination observed in *B. pseudomallei* can introduce new genetic variations, thereby enabling detection of selection signatures in other contemporary populations outside Australia (Chewapreecha et al., 2019). Natural selection on modern humans as a result of pathogen encounter and migration has been reviewed elsewhere (Karlsson et al., 2014; Sironi et al., 2015; Slodkowicz and Goldman, 2020).

**FIGURE 1:**
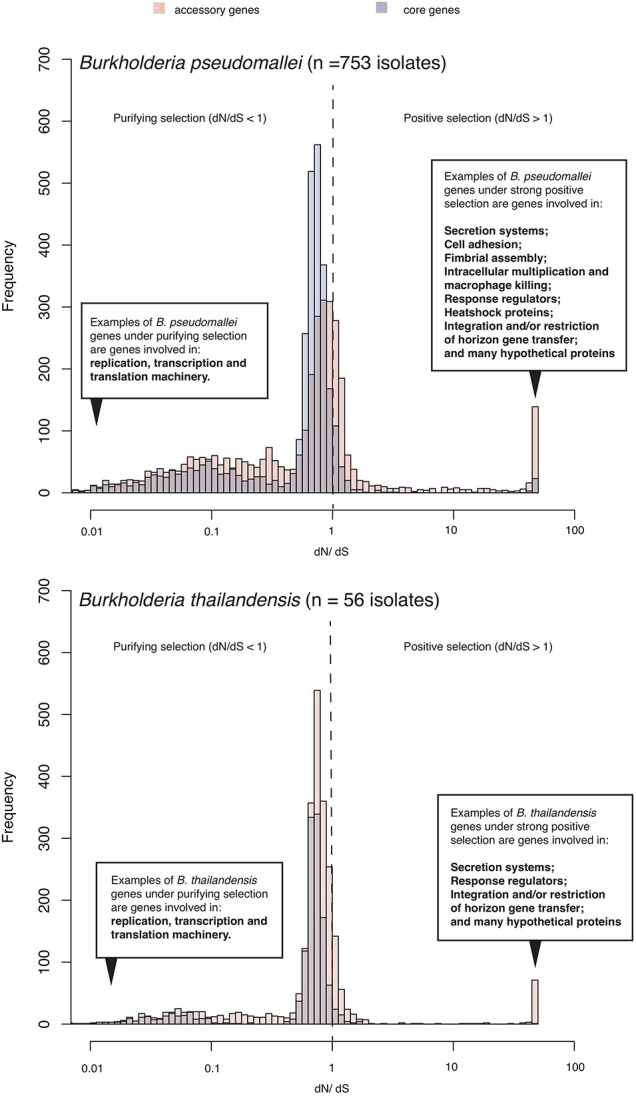
Selection pressure acting on *B. pseudomallei* population. The histogram summarizes ranges of *dN/dS* calculated from predicted coding sequences from a collection of diverse *B. pseudomallei* population from northeast Thailand (Chewapreecha et al., 2019), and *B. thailandensis* genomes from the public database. *B. pseudomallei* and *B. thailandensis* have highly plastic genomes comprising of at least two chromosomes of ∼7–8Mb in size when combined. Using a pan-genome approach, all coding sequences could be categorized as “core” (present in all genomes) or “accessory” (variably present across studied genomes). Accessory genes display an elevated level of *dN/dS* which is signatures of positive selection or more relaxed purifying selection.

For bacteria, evolutionary pressures on orthologous proteins can be quantified using the ratio between substitution rates at non-synonymous (*dN*) sites, which could have experienced selection, and synonymous (*dS*) sites which are presumably neutral. The *dN/dS* ratio is likely to be more than 1 if pressures favor changes in the protein sequence (positive selection). It is likely to be less than 1 if selections suppress changes in the protein sequence (purifying selection). Several studies (Yu et al., 2006; Losada et al., 2010; Nandi et al., 2010; Hayden et al., 2012; Chewapreecha et al., 2019) and this review utilized *dN/dS* to estimate selection pressures on coding sequences of the pathogenic *B. pseudomallei* as well as non-pathogenic but closely related species such as *B. thailandensis*. Although these studies focused on bacterial populations isolated from different melioidosis endemic regions, performed on different sample size and genetic diversity, they largely highlighted three similar patterns (Figure 1). The first common pattern is an elevated level of positive selection in *Burkholderia* accessory genes (genes that are variably present across different isolates) compared to core genes (genes that are consistently present in all isolates). This observation highlights the role of accessory genomes in mediating *B. pseudomallei* adaptation, a feature reflected by the open pangenome and large repertoire of accessory genes observed in *B. pseudomallei* and *B. thailandensis* (Nandi et al., 2015; Spring-Pearson et al., 2015; Chewapreecha et al., 2017). The second common pattern is the set of genes under purifying selections in *B. pseudomallei* and *B. thailandensis* involved in the bacterial replication, transcription and translation machinery; highlighting the conservation of these genes in both species. The third common pattern is that many genes under positive selection in *B. pseudomallei* and *B. thailandensis* are required for environmental survival and exchange of genetic materials including secretion systems, response regulator, heat shock proteins, and integration and/or restriction of horizontal gene transfer. However, genes required for host cell invasions such as cell adhesion, fimbriae, intracellular multiplication and macrophage killing are only positively selected in *B. pseudomallei* but not *B. thailandensis*. These distinct signals for positive selection in *B. pseudomallei* and *B. thailandensis* could mark a different pathogenic potential of the two species.

Genes under positive selection could primarily offer *B. pseudomallei* advantages to survive harsh environmental conditions. Given its tropical and sub-tropical habitats, *B. pseudomallei* experiences drought and heavy rainfall on a seasonal basis. The bacterium can be found in deeper soil layers during the dry season and moves to the soil surface during the rainy season following water movement (Thomas et al., 1979; Currie and Jacups, 2003; Manivanh et al., 2017), highlighting a spatiotemporal transition. The bacterium is also subjected to heat stress. An *in vivo* study showed that *B. pseudomallei* had a slower growth rate and a shift in gene expression toward heat-shock proteins and bacterial motility when exposed to temperature stress (Paksanont et al., 2018). Intriguingly, *B. pseudomallei* is capable of persisting in a nutrient-free distilled water (Pumpuang et al., 2011). The bacterium was subjected to distilled water since 1994 and this experiment is still ongoing, thereby marking an unusual ability of *B. pseudomallei* to survive in low nutrient media and high osmotic pressure. Lipopolysaccharide, a known virulence factor, was proposed to facilitate *B. pseudomallei* survival in water. Statistical analysis suggested that there is a correlation between the presence of LPS and rainfall, in particular, LPS serotype B (Shaw et al., 2019). An experimental study further reported that a gene of inner core LPS biosynthesis cluster (*waaE: BPSL2510)* is vital for a long-term water incubation (Moore et al., 2008). Moreover, an adhesin *BPSL1661* was identified as a hub for co-evolutionary signals in *B. pseudomallei* population (Chewapreecha et al., 2020). The gene was functionally characterized as essential during nutrient starvation (Chewapreecha et al., 2020), thereby highlighting nutrient limitation as a major evolutionary pressure experienced by this microorganism. The presence of *B. pseudomallei* is strongly associated with the low-nutrient soil (Limmathurotsakul et al., 2010b; Hantrakun et al., 2016). This led to the hypothesis that *B. pseudomallei* might have a competitive advantage over benign soil microbes in nutrient-depleted soil but is outcompeted in a nutrient-rich environment. The occurrence of *B. pseudomallei* in the nutrient-poor agricultural field has linked to crop residue burning, a common practice in the tropics where the burning practice could deplete the soil organic matter (Bot and Benites, 2005). An ability to persist in the hostile environmental conditions allows the bacterium to survive before it could seek shelters in a more protecting reservoir inside the single or multicellular hosts.

Genes that facilitate bacterial transition from the environment to hosts are also under positive selection. A genome-wide association study (GWAS) recently identified the bacterial genetic factors that distinguish between *B. pseudomallei* isolated from the environment and those causing disease in human (Chewapreecha et al., 2019). Genetic variants associated with cell entry and toxin were found to be more prevalent in disease- than environmental isolates, while variants involved in malfunctional cell adherence were found at higher frequency in environmental- than disease isolates. Together, this highlights significant roles of cell adhesion and cell entry in allowing the bacterium to switch to an intracellular lifestyle, either in an amoeba or in a human host where it causes melioidosis. The same study also quantified the numbers of time the variants were gained or lost from the population phylogenetic tree and highlighted multiple gain-and-loss events for both disease- and environment-associated variants. This genetic evidence suggests a process into which *B. pseudomallei* can adapt to colonize multiple niches by exploiting existing variations or exchanging disadvantageous for advantageous alleles that promote its survival in a new niche, including the human host.

## Mechanisms That Generate Genetic and Molecular Variations

Genetic information in both *B. pseudomallei* and human host can be passed on vertically, although mistakes during replication process could result in small-scale genetic variations. In *B. pseudomallei*, larger-scale genetic variations can be introduced by recombination event or horizontal gene transfer (HGT). The former process is akin to sexual reproduction and meiotic crossing-overs in its human hosts. Due to contrasting short- and long- generation time of *B. pseudomallei* (49 min in the log phase; Ou et al., 2005) and host (22–33 years for modern humans; Ewbank, 2016); the host cannot solely rely on new genetic diversity generated when the new offspring is born. A healthy human possesses a large and dynamic repertoire of B cell- and T cell receptors that recognize the invading pathogen and produce a repertoire of antibody that recognizes a variety of antigenic structure. This molecular diversity is achieved without a change in genetic content. The mechanisms that generate the genetic and molecular diversity of the human immune system have been reviewed elsewhere (Nikolich-Zugich et al., 2004; Robinson, 2015; Dendrou et al., 2018; van den Broek et al., 2018; Adams et al., 2020).

Small-scale genetic variations in *B. pseudomallei* can be introduced by point mutation which substitutes one nucleotide with another or microindel (an insertion or deletion) that impact 1–50 bp. *B. pseudomallei* substitution rate was reported to be 1.7–4.9 × 10^–7^ substitutions per site per year (Viberg et al., 2017; Pearson et al., 2020), which is comparable to those detected in other *Burkholderia* genera (Lieberman et al., 2014). The rates of indels have not been quantified in *B. pseudomallei*. However, indels can be detected from isolates obtained from acute and chronic host infections (Hayden et al., 2012; Limmathurotsakul et al., 2014a; Viberg et al., 2017) as well as from the environment (Rachlin et al., 2020), thereby highlighting the role of indels in *B. pseudomallei* evolution.

Medium-scale genetic variations can be brought in by homologous recombination, with each event contribute to a median recombining size of 5 kb (Nandi et al., 2015) (range 3 bp to 71 kb). A single recombination event can introduce 7.2 times greater nucleotide polymorphisms than a single substitution event (average r/m = 7.2). However, the amounts of SNPs being introduced per recombination event is subjected to the genetic distance between DNA donor and recipient. Sources of imported DNA could be from the same species donor, closely related species such as *B. thailandensis* and/or other soil microbes. Different *B. pseudomallei* lineages have been shown to recombine at different rates (Nandi et al., 2015), a characteristic also observed in other recombinogenic bacteria (Chewapreecha et al., 2014; Sanchez-Buso et al., 2014; David et al., 2017). Interestingly, not all DNA donor-recipient pairs are possible, suggesting a structure to the genetic flux within *B. pseudomallei* population. A lineage-specific restriction-modification system was shown to act as a barrier that restricts gene flow (Nandi et al., 2015). These modification control systems are based on DNA methylation which allows the bacterium to discriminate between correctly methylated “self” DNA, and inappropriately methylated or unmethylated “non-self” DNA. Correctly methylated DNA can be taken up and integrated into the bacterial genome, whereas inappropriately methylated or unmethylated DNA will be degraded. Interestingly, many recombination “hotspots” are focused on bacterial virulence factors (Nandi et al., 2015), highlighting the role of recombination in tuning the bacterial ability to infect hosts.

Large-scale genetic variations can be introduced by HGT or large-scale insertion or deletion. HGT includes the uptake of foreign DNA that can subsequently be integrated into the chromosomes and resulted in the regions termed genomic islands (GIs) (Koonpaew et al., 2000; Holden et al., 2004; Duangsonk et al., 2006). The repetitive genetic composition of *B. pseudomallei* genomes such as tandem repeats and transposons is believed to aid the integration of horizontally acquired elements into the chromosomes (Holden et al., 2004; U’Ren et al., 2007). Despite several plasmid elements being identified in *B. pseudomallei* genomes (Holden et al., 2004), four complete plasmids has been characterized so far (NCBI, December 2020) (Nandi et al., 2015). With long-read sequencing technology, the plasmid could be more reliably assembled, thereby allowing more genetic variations to be studied. At least 16 GIs had been identified in the K96243 genome (Holden et al., 2004). Several studies highlight distinct geographical distribution of GIs or their combinations (Ou et al., 2005; Duangsonk et al., 2006; Sim et al., 2008; Tuanyok et al., 2008), leading to a hypothesis that each GI or combination of GIs may confer different fitness under different environments (Sim et al., 2008). GIs carry several virulence factors, many of which are known to interact with hosts, including filamentous hemagglutinin adhesin (*fhaB3*), and a member of two-partner secretion system (*bpaAB*) (Sim et al., 2008).

## Genetic, Structural or Molecular Variations Observed in Host-Pathogen Interaction

**FIGURE 2:**
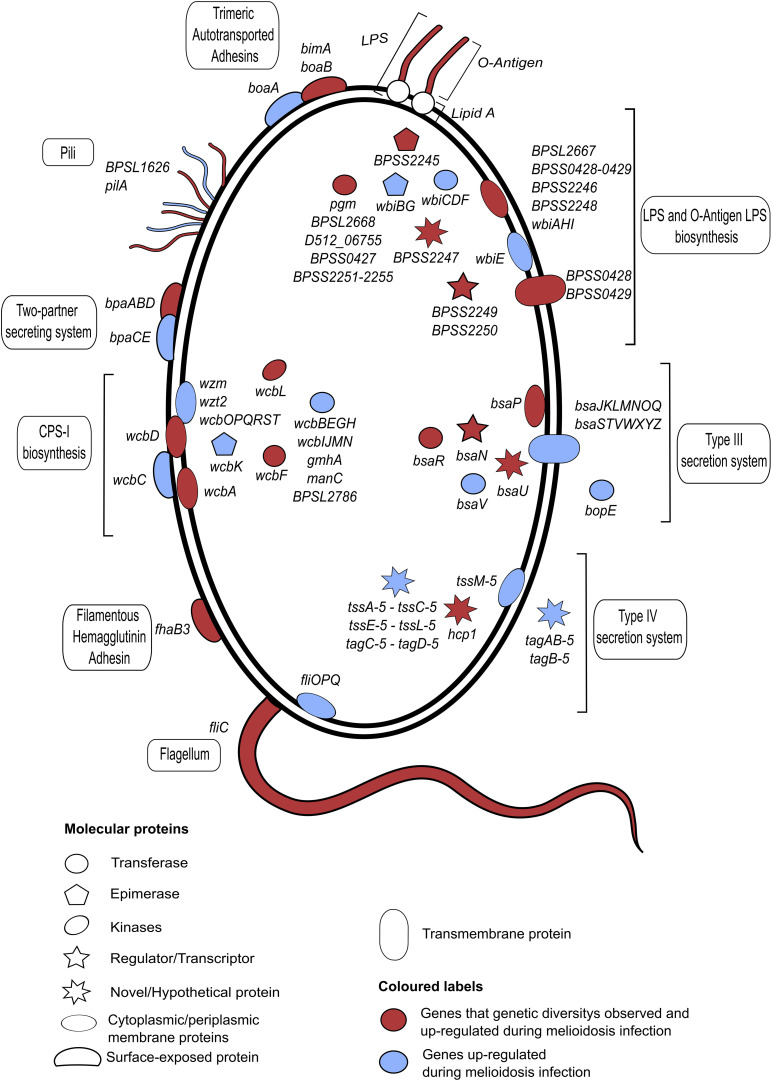
A summary of *B. pseudomallei* genes or operons that are expressed during infection and characterized genetic variations. All plotted genes or operons are up-regulated during *in vivo* infections (Ooi et al., 2013), with a subset that reported genetic variations marked in red. The shape and location of each individual gene indicate the gene function and cellular compartment, respectively. All annotated genes or operons and their functions are described. The bacterium displays a repertoire of antigenic variations, including lipopolysaccharides (LPS), capsular polysaccharides (CPS) and surface proteins. *B. pseudomallei* LPS is immunologically classified into a number of serotypes A, B, and B2; with each serotype reported to be heterogeneously distributed across distinct geographical locations. Another highly diverse virulent protein is a fimbrial protein which displays a strong geographical distribution between Australia and Asia. Strains from Asia commonly possess a *Yersinia*-like fimbrial (YLF) gene cluster that believed to be horizontally acquired. *B. pseudomallei* carries 4 different types of CPS: CPS I, CPS II, CPS III, and CPS IV. A full gene description is provided in Supplementary Table 1.

**FIGURE 3:**
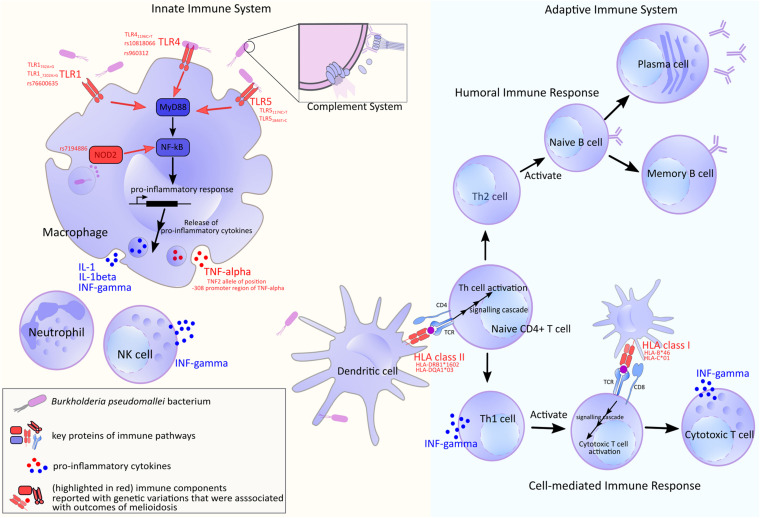
A summary of human host immune components used in defending against *B. pseudomallei.* Genes or molecules that show genetic variations are marked in red. Upon infection, the bacterial antigens are recognized by host receptors including Toll-like receptor (TLR) and HLA which also display large genetic diversity. The latter is reported to be varied by ethnic groups (de Bakker et al., 2006; Gourraud et al., 2014). Following an entry into host, *B. pseudomallei* faces the innate axis of the immune system. Macrophages and neutrophils are recruited to the site early upon the infection; these cells are reported to be essential to the early bacterial containment and clearance (Breitbach et al., 2006; Easton et al., 2007), though excessive recruitment of neutrophils may have a negative outcome that allows *B. pseudomallei* to propagate intracellularly (Ceballos-Olvera et al., 2011). These innate phagocytes possess pattern recognition receptors (PRRs), such as surface receptors TLR2, TLR4, and TLR5, as well as, cytosolic receptors NOD2; these are reported to be vital for the fight against *B. pseudomallei* (West et al., 2008; Myers et al., 2014; Weehuizen et al., 2015; Birnie et al., 2019). The signals transduced by these receptors result in mobilization of nuclear factor NF-κB which trigger appropriate immune responses including the synthesis of pro-inflammatory cytokines and initiation of the downstream adaptive immune cascades (Pothlichet and Quintana-Murci, 2013). It is through the antigen-presenting cells and their appropriate antigen-HLA class II complexes that enable the activation of CD4 + T lymphocytes. The naïve CD4 + T cells sit at the central part of the adaptive axis. They can differentiate into Th1 cells which facilitate cell-mediated immune response by CD8 + cytotoxic lymphocytes. A study has shown that strong CD4 + and CD8 + T cell response was elicited during acute melioidosis and the lower cellular response was correlated to fatality (Jenjaroen et al., 2015). On the other hand, naïve CD4 + T cells can also mature to be Th2 cells which initiate class switching of B cells and support humoral immune response.

Genetic, structural and molecular variations and their functional impact to *B. pseudomallei* - host interaction have been the center of melioidosis research over the past few decades. We will summarize genetic and structural diversity in bacterial genes known to mediate the infection (Figure 2 and Supplementary Table 1). The genes discussed here are shown to be essential for infection by a genome-wide saturation mutagenesis (Moule et al., 2015) and also expressed during *in vivo* infection (Ooi et al., 2013). When host partners are known; variations detected in host proteins, and the host response are also described (Figure 3). It should be noted that there are large overlaps in the host immune system and the examples described here only represent a fraction of the whole machinery.

### Genetic Variations in *B. pseudomallei* Flagella Systems and Host Toll-Like Receptor 5 (TLR5)

Flagella systems of *B. pseudomallei* enable the motility of the bacterium intracellularly as well as in the environment (Figure 2; DeShazer et al., 1997; French et al., 2011). Flagella filaments are constituted from thousands of flagellin protein monomers, encoded by *fliC* gene, strung into protofilaments before being braided to form a flagellum (Samatey et al., 2001). Flagellin has been known for its extreme diversity with 113,285 unique nucleotide sequences across the prokaryote phyla (Hu and Reeves, 2020). Flagellin inactivation in *B. thailandensis* resulted in the reduction of bacterium intracellular motility and cell-to-cell spread (French et al., 2011). Using PCR-restriction fragment length polymorphism analysis, four different alleles of flagellin protein (*fliC: BPSL3319*) were identified from 100 Malaysian clinical isolates (Tay et al., 2010), and more is expected if investigated with next-generation sequencing. Proteins in the flagella systems were reported to elicit host immune response, highlighting them as melioidosis vaccine candidates (Brett et al., 1994; Chua et al., 2003; Chuaygud et al., 2008; Gregory et al., 2015; Koosakulnirand et al., 2018).

In addition to flagella, a fimbrial gene cluster which is required for cell adherence also displays genetic variation with distinct alleles being predominantly detected in different geographical locations. A *yersinia*-like fimbrial (YLF) gene cluster, believed to be horizontally acquired was shown to be more prevalence in Southeast Asia and thus was used as a marker for the introduction of *B. pseudomallei* from this region (Tuanyok et al., 2007; Sarovich et al., 2014; Chewapreecha et al., 2017). A putative type I fimbrial protein *BPSL1626* was shown to elicit an immune response and have a potential as a vaccine candidate against melioidosis (Capelli et al., 2018). Whereas, *B. thailandensis-*like flagellum and chemotaxis (BTFC) gene cluster is believed to be an ancestral sequence in *B. pseudomallei* and is most common in Australia (Tuanyok et al., 2007). The YLF and BTFC gene clusters are mutually exclusive between the two endemic areas where the latter may implicate in the spread between cell-to-cell by the flagellar protein leading to the formation of multinucleated giant cells (MNGCs) and eventually apoptosis/cell death (French et al., 2011).

It has been established that flagellin is a ligand of TLR5 (Figure 3). The recognition stimulates pro-inflammatory responses including the rise in intracellular calcium ion and upregulation of pro-inflammatory cytokine TNF-α and IL-6 (Hayashi et al., 2001; Chen et al., 2007). TLR5 stop codon polymorphism, *TLR5*_117__4C__>__*T*_ or rs5744168 was strongly associated with protection against fatality as well as organ failure in a case-control cohort study. When challenged with *B. pseudomallei, TLR5*_117__4C_ could mediate the activation of NF-κB, while *TLR5*_117__4T_ could not; additionally, *TLR5*_117__4T_ saw reduced flagellin-induced cytokines levels (West et al., 2013). Another independent investigation on the same population also found the association between the truncated *TLR5*_117__4C__>__*T*_ variant and survival from acute melioidosis, as well as, a lower rate of bacteraemia (Chaichana et al., 2017). Furthermore, both studies also found *TLR5*_117__4C__>__*T*_ variant with a lower level of anti-inflammatory IL-10, which the authors suggested the possibility in which the mortality risk may be modulated by TLR5-driven IL-10 release. Interestingly, a recent study has suggested that the effect of this hypofunctional TLR5 variant may not be restricted to flagellin-driven pathway (Dickey et al., 2019). Another polymorphic variant is *TLR5*_184__6T__>__*C*_ which was also associated with protection against death and blunted flagellin-driven cytokine response; however, the authors also reported high linkage disequilibrium of the variant with TLR51174C>T (Chantratita et al., 2014), which might reduce the confidence of causal relationship.

### Structural Variations in *B. pseudomallei* LPS and Genetic Variation in Host Toll-Like Receptor 2 and 4 (TLR2 and TLR4)

*B. pseudomallei* possesses an extensive network of polysaccharides on its outer membrane, namely capsular polysaccharide (CPS) and lipopolysaccharide (LPS) (Figure 2, for a full review on CPS and LPS in *Burkholderia* spp.; Cloutier et al., 2018). Both are known to play a vital role in virulence of melioidosis and have been used as subunits for vaccine development (Brett and Woods, 1996; Nelson et al., 2004; Wikraiphat et al., 2009; Scott et al., 2014b). Based on a cellular compartment, *B. pseudomallei* LPS can be divided into (Knirel et al., 1992): lipid A—an endotoxic component embedded in the phospholipid bilayer of the outer membrane; inner and outer core oligopolysaccharide; and O-antigen (Novem et al., 2009). Multiple *in vitro* studies showed LPS challenge could mount innate and adaptive immune responses, and Nitric Oxide (NO) production, with different LPS serotypes (A, B, and B2) reported to mount different magnitude of responses (Norris et al., 2017). Some genetic data of LPS biosynthesis operons were available. However, many of these were generated from short-read sequencing platforms, which are not ideal to investigate LPS due to the repetitive nature of the LPS locus. Long-read technologies, on the other hand, could overcome this assembly issue, giving complete contigs of the bacterium genome.

To date, 4 serotypes of LPS were previously characterized. Type A LPS is a majority serotype found in both endemic areas, namely Southeast Asia and Australia (Anuntagool et al., 2000, 2006; Tuanyok et al., 2012). Whereas, serotype B is less common in the endemic areas (Australia and Southeast Asia) and is prevalent in both clinical and environmental origins (Tuanyok et al., 2012). However, a recent analysis of clinical *B. pseudomallei* isolates revealed that Serotype B was highly predominant in India (Shaw et al., 2019). LPS serotype B2 is a variant of LPS serotype B and more commonly found in Australia and Papua New Guinea (Tuanyok et al., 2012). In contrast, LPS R or Rough serotype lacks O-antigen moiety of the LPS structure. It was identified using SDS-PAGE and silver staining techniques with no O-antigen ladder pattern. Type R is relatively rare and found only from the Australian clinical and environmental strains (Anuntagool et al., 2006). Interestingly, it is frequently prevalent in the patients with a relapse history of melioidosis, however, there is no direct association between them (Limmathurotsakul et al., 2014b).

A comparative genomic analysis revealed that there are distinct variations in the core compositions of the O-antigen LPS biosynthesis gene clusters between A, B, and B2 serotypes. However, there are several genes conserved among them. In addition, type A LPS gene operon is also observed in both *B. thailandensis* and *B. mallei*, closely related *Burkholderia* species (Tuanyok et al., 2012). Interestingly, genomic analysis of clinical isolates from Madagascar revealed that there is a 13.5 kb deletion observed in the LPS biosynthesis gene cluster of serotype B, conserving only some genes in the cluster that are essential for the biosynthesis of LPS B2 (Sarovich et al., 2016). The lack of some core genes in the LPS biosynthesis cluster could lead to the reduction of its serological properties. This is supported as some evidence suggested that the strains with LPS type B2 become sensitive to 30% normal human serum whereas the strains with LPS type B remain resistant (Tuanyok et al., 2012).

On the other hand, a single nucleotide insertion of the *wbiI* gene is observed in the LPS biosynthesis gene cluster of a rough serotype in a patient with more than 16-year chronic lung infection associated with melioidosis. This frame-shift mutation of *wbiI* gene (an essential gene for the O-antigen synthesis) disrupts the epimerase/dehydratase function of this gene and results in the loss of O-antigen moiety, possibly switching the serotype of *B. pseudomallei* isolates from type A to type R (Price et al., 2013; Pearson et al., 2020). Pearson and colleagues also revealed the nucleotide insertion of *D512_15771 (wbiH)* and *D512_06755* gene are observed in *B. pseudomallei* MSHR6686 of the same patient. The mutation in both genes may confer to the reduction in LPS modification and production, assisting in the escape from the host immune response. In addition, a partial deletion of *D512_20407, wbiA* homolog is also identified in MSHR6686 (Price et al., 2013; Pearson et al., 2020). However, genomic analysis of the rough serotype that is naturally found in the environment or initial infection has not been done to determine the genetic makeup of this serotype.

Not only genetic heterogeneity exists among the serotypes of LPS, further structural diversity is also observed at the O-antigen of LPS serotypes (Stone et al., 2012; Norris et al., 2017). The O-antigen is one of the LPS components with a structure of unbranched disaccharide repeat units. Remarkably, the structural modifications of these sugar chains were observed where substitutions of 2-O-methylated and 4-O-acetylated at talose residues were observed only in about 33% of the LPS serotype A. Whereas the rest bear 2-acetyl substituents at the same residues (Perry et al., 1995). More recently, a structural analysis of O-antigen in the serotype A reported that the modification of the talose residue is more complex than what was previously reported (Heiss et al., 2013). Although multiple gene inactivation studies revealed that *wbiA* and *oacA* genes are essential for these modifications at the talose residues (Brett et al., 2003, 2011), it is possible that structural diversities of the LPS O-antigen are further modified at post-transcriptional and post-translational levels, for instance, a length variation of O-antigen chain observed in *Escherichia coli* O9a which is tightly controlled by the biosynthetic enzymes dynamic (King et al., 2014).

The LPS is common across Gram-negative bacteria and also a well-established pathogen-associated molecular pattern (PAMP) that can trigger the pro-inflammatory innate immune response, such as translocation of NF-κB and TNF-α cytokine release, via its interaction with TLR4, CD-14 and adaptor protein MD-2 (Park and Lee, 2013). Although evidence has been inconclusive, LPS sensing was shown to be through TLR4 in murine models, and TLR4 as well as TLR2 (Wiersinga et al., 2007; West et al., 2008) in human models (Figure 3; Weehuizen et al., 2015). Polymorphisms of TLR4 were reported in humans with *TLR4*_119__6C__>__*T*_ allelic variant associated with protection against melioidosis when compared to non-hospitalized controls (West et al., 2012). In addition, TLR4 rs10828066 SNP variant was significantly associated to be protective against melioidosis in both adjusted non-hospitalized and hospitalized control groups, whereas TLR4 rs960312 was associated with bacteraemic or lung melioidosis (West et al., 2012).

### Genetic Variations in *B. pseudomallei* CPS and Host B Cell Repertoire

CPS is one of the key virulence determinants in *B. pseudomallei* (Figure 2). To date, four different types of CPS (CPS I-IV) have been described (Holden et al., 2004). CPS-I biosynthesis cluster is a large 34.5 kb operon harboring on the chromosome one of *B. pseudomallei* (*wcbT* to *manC*). A study in an animal model reported that the CPS-I cluster is required for the full virulence of *B. pseudomallei* (Sarkar-Tyson et al., 2007). CPS-I exhibits an immunogenic role and the absence of this gene cluster resulted in the attenuation of *B. pseudomallei* in a mouse model (Atkins et al., 2002; Reckseidler-Zenteno et al., 2005; Parthasarathy et al., 2006). A passive protection was observed when murine intranasal infection models were immunized with anti-CPS monoclonal antibodies (AuCoin et al., 2012), with the later study reported that conjugated CPS could provide the highest degree of protection (Scott et al., 2014a).

The structural basis of CPS-I is highly conserved (Perry et al., 1995). Remarkably, CPS-I is believed to be horizontally transferred between species as the GC content of this CPS-I cluster is about 58% (Reckseidler et al., 2001). In addition, it is possible that the horizontal acquisition of this gene cluster was a key event in the pathogenic evolution of *B. pseudomallei* compared to *B. thailandensis* where the cluster is absent (Yu et al., 2006). Nevertheless, several genes involved in the biosynthesis of CPS-I of *B. pseudomallei* were reported in *B. thailandensis* with a relatively low sequence identity (75%). In addition, a novel variant of *B. thailandensis* strains (BTCV) is found to carry a *B. pseudomallei* like CPS-I (95% sequence identity) gene cluster with an identical organization. Although the origin of the CPS-I gene cluster in *B. pseudomallei* has yet been identified, several genes involved in the sugar biosynthesis appear to be orthologs to the genes identified in the Yersinia species *(Y. pseudotuberculosis H892/87)* and some in other gram-negative bacteria (Cuccui et al., 2012). It is possible that the CPSI gene cluster is not entirely acquired from one bacterial species but several organisms. Population studies also detected genetic variations in CPS-I (Challacombe et al., 2014; Chewapreecha et al., 2017). Whether this genetic variation has implications on virulence is a subject of further investigation. Another CPS-I variant could be found in *B. pseudomallei* isolated from a 16-year chronic melioidosis patient. A single nucleotide insertion is observed within *wcbR*, an important component involved in the fatty acid synthesis of CPS-I, causing a frameshift mutation in the earlier samples isolated from this patient (Price et al., 2013; Pearson et al., 2020). A CPS-I deletion region, which includes *wcbR* gene, has shown in the reduction of CPS production in hamster model but not absence entirely (Gutierrez and Warawa, 2016). Price et al. (2013), suggested that this degree of CPS-I-dependent virulence decreases and may consequently be a critical step in the progression for melioidosis to become a chronic-carriage disease.

Unlike CPS-I, CPS-II and CPS-III have been associated with *B. pseudomallei* persistence in the environment (Reckseidler-Zenteno et al., 2009, 2010), while CPS-IV is less well-characterized. Up to date, no reports suggest the genetic variations of CPS-II, -III, and -IV biosynthesis clusters in *B. pseudomallei.* The diversity and function of these capsules in the pathogenesis, immunomodulation and environmental adaptation of *B. pseudomallei* warrant further studies.

We next considered host partners that interact with *B. pseudomallei* CPS. The CPS has been shown to elicit strong host immune response (Reckseidler et al., 2001; Atkins et al., 2002). CPSs from various *B. pseudomallei* strains were recognized by the same group of monoclonal antibodies which suggests a limited number of epitopes of this molecule (Zou et al., 2008). The molecule’s monomers can cross-link to B cell receptors and, at an appropriate density, induce a downstream humoral response that is distinct from T cell-dependent pathway (Clarke et al., 2013; Akkaya et al., 2020). Antigen recognition through B-cell receptors is formed through random somatic changes of germline DNA. This results in a repertoire of distinct sequences that enable antigen recognition across wide ranges of pathogens. The B-cell receptors dynamic in melioidosis has not been studied.

### Genetic Variations in *B. pseudomallei* Adhesins and Autotransporters

Filamentous Hemagglutinin Adhesin (FHA) of *B. pseudomallei* is highly diverse (Figure 2). Early investigation of genomic islands (GIs) from five *B. pseudomallei* strains identified three different *fhaB* gene clusters on different GIs of the bacteria. The bacterium could carry multiple *fhaB* gene clusters, many of with carrying either a combination of cluster I (GI5a/GI5a.1/GI5a.2) and cluster III (GI16/GI16.1), or cluster III alone (Tuanyok et al., 2008). More genetic variations of the FHA loci were reported from the Australian isolates (Chewapreecha et al., 2017). The *fhaB3* gene (*BPSS2053*) has been characterized as an important virulence factor of *B. pseudomallei*; enabling bacterial binding to the host epithelial cells (Sim et al., 2008), and an anti-macrophage factor (Dowling et al., 2010). It was found in all isolates from Thailand but found in only 83% of Australian strains where the absence of this gene in Australia population correlated with the skin abscess formation and lower mortality rate (Sarovich et al., 2014).

Autotransporter (AT) proteins are one of the largest family of the secretion systems in Gram-negative bacteria, allowing the transportation across the bacterial membrane as well as involving the virulence and immunogenicity of the pathogens (for review Lazar Adler et al., 2011). In *B. pseudomallei* K96243, a sequence analysis revealed to have at least 11 ATs located in the genome, including putative Trimeric Autotransporter Adhesins (TAAs), *bimA* (Stevens J.M. et al., 2005) and *boaB*, two-partner secretion system (TPS), *bpaA, bpaB*, and *bpaD* (Campos et al., 2013). Inactivation in some of these genes attenuated and reduced the intracellular survival of *B. pseudomallei* in the macrophage-like cells (Lazar Adler et al., 2015). ATs are variably present in the genomes. The gene *boaB (BPSL1705)*, which aids bacterial binding to host respiratory cells (Balder et al., 2010), was found to be absent in several strains in Africa, Brazil (Sarovich et al., 2016). When ATs are present, genetic variants can also be observed. Australian-specific and African-specific variants have been reported in *bpaA, bpaB*, and *bpaD (BPSS1434, BPSL2063*, and *BPSS0088*, respectively) (Brown et al., 2004; Tuanyok et al., 2008).

Several experiments demonstrated the role of BimA in actin polymerization and motility (Stevens M.P. et al., 2005; French et al., 2011), promoting a movement of cell-to-cell spread and within the host cells (Lazar Adler et al., 2011). Furthermore, BimA is antigenic (Suwannasaen et al., 2011) and seroactive (Felgner et al., 2009). Two variants of *bimA* (*BPSS1492*) were identified: *B. pseudomallei bimA* (*bimA*_*BP*_), and *bimA*-like *B. mallei (bimA_*BM*_)*. The latter is an ortholog of *bimA* from *Burkholderia mallei* (95% sequence identity) and displays the same domain organization: a single actin monomer binding motif (WH2), a proline-rich domain and a transmembrane anchor domain (Sitthidet et al., 2008). Whereas the *bimA*_*BP*_ consists of two predicted WH2 domains, a proline-rich domain, a membrane anchor domain and an additional predicted casein kinase II (Stevens M.P. et al., 2005). Of note, the variation was also observed between the predicted proline-rich domain of the *bimA*_*BM*_ and *B. mallei bimA* where the former has fewer motifs (Sitthidet et al., 2008). Distinct geographical distribution of *bimA*_*BP*_ and *bimA*_*BM*_ have been reported (Sitthidet et al., 2008; Sarovich et al., 2014; Shaw et al., 2019). Benanti et al. (2015) illustrated that both BimAs are functionally similar and associated with the nucleation and elongation of actin filaments with more plaque formation observed in *bimA*_*BP*_ variant compared to *bimABM*. A subunit vaccine investigation reported a significant increased survival rate of BALB/c mice immunized with BimA_*BM*_ recombinant, when the mice were challenged with both *B. mallei* and *B. pseudomallei* (Whitlock et al., 2010).

### Genetic Variations in *B. pseudomallei* Secretion Systems

Several secretion systems and secreted proteins in *B. pseudomallei* are vital to combat the host immune defense and environmental stresses. *B. pseudomallei* has at least three type III secretion systems (T3SSs) (for review, Vander Broek and Stevens, 2017)and six type VI secretion systems (T6SSs).

Among T3SSs, T3SS-3 is better characterized and believed to be an integral part of the full *B. pseudomallei* virulence in mice and hamster models, as well as associated with the intracellular survival and dissemination. K-mer based sequence analysis has recently demonstrated that variations in the T3SS-3 gene cluster were detected across the global population of *B. pseudomallei* (Chewapreecha et al., 2017). This included *bsaU(BPSS1539), bsaR (BPSS1542), bsaP (BPSS1544), and bsaN* (BPSS1546) (Chewapreecha et al., 2017) which are believed to be involved in intracellular escape (Pilatz et al., 2006), predicted chaperone protein (Panina et al., 2005), T3SS-3 secretion regulator (Broek et al., 2015), and T3SS-3 regulator (Chen et al., 2014), respectively. In addition, a partial deletion of genes in the T3SS is also observed in a patient with persisted infection of melioidosis as a result of within-host adaptations (Pearson et al., 2020). For T6SSs, T6SS-5 is better characterized (for review, Lennings et al., 2019)and functionally confirmed to mediate the translocation of effector proteins via contact-dependent manner (Silverman et al., 2012) and MNGC formation (Burtnick et al., 2011; Chen et al., 2011). In addition, an experimental study suggested that T6SS-5 may involve in the intracellular survival of *B. pseudomallei* in macrophages where the expression level of T6SS-5 is dependent on *virAG and bprC* regulatory gene (Chen et al., 2011). Genetic variations are also observed in several genes clustered T6SS-5 with distinct geographical distribution (Chewapreecha et al., 2017). Remarkably, a large deletion region on chromosome 2 of an environmental *B. pseudomallei* A4 and an isogenic strain of K96243 has been demonstrated of which they failed to form a plaque in epithelium cells. Of those, the deleted genes are including genes involved in T3SS-3 and T6SS-5 systems. Interestingly, Saiprom et al. (2020) also identified an absence of multiple T3SS-1 genes of previously described Thai environmental strain RF80.

### Genetic Variations in Human Leukocyte Antigen (HLA)

For the rest of the bacterial factors that do not get recognized by any specific innate pattern recognition receptors, these antigens will be processed and presented on HLA of antigen-presenting cells such as dendritic cells before getting recognized by appropriate T cell receptors (TCR) of the adaptive immune axis; this HLA-peptide-TCR interaction kicks start the adaptive immune response. There are three different classes of HLA: class I interacts with TCRs on CD8 + T cells while class II binds to TCRs on CD4 + T cells, and the less well-established class III which is not involved in antigen processing and presentation (Dendrou et al., 2018). Acute melioidosis patients with diabetes mellitus were reported to have lower HLA-DR expression on plasmacytoid dendritic cells than the non-diabetic diseased group (Kronsteiner et al., 2019). In addition, in non-diabetic patients, fatal cases presented with significantly lower expression of HLA-DR on monocytes and plasmacytoid dendritic cells, compared to the survived cases (Kronsteiner et al., 2019). Similar observation was found in murine infection models where *B. pseudomallei-*infected BALB/c and C57BL/6 mice showed reduced expression of MHC class II on plasmacytoid dendritic cells, however, this was not statistically significant (Williams et al., 2015).

Genetic variations in HLA have been linked to the melioidosis outcomes. A work conducted in an endemic area of Thailand has compared HLA allele frequencies in melioidosis cases and healthy controls. The authors reported a significant increase of DRB1^∗^1602 frequency in melioidosis patients, compared to healthy controls. Moreover, an increase in HLA-DRB1^∗^1602 and a decrease in HLA-DQA1^∗^03 allele frequencies were associated with septicaemic cases of melioidosis (Dharakul et al., 1998). Another study conducted on the same Thai population screened a panel of various HLA class I genotype frequencies in survived and fatal cases of melioidosis. The authors found that HLA-B^∗^46 and HLA-C^∗^01 were associated with increased mortality; they were also reported to be in linkage disequilibrium (Dunachie et al., 2017). Interestingly, HLA has been linked with diabetes mellitus, the prominent comorbidity of melioidosis with 12-fold increased risk of developing the disease (Limmathurotsakul et al., 2010a). Several recent publications using GWAS have identified variations of HLA and their corresponding protective or predisposing association with type 2 diabetes (Williams et al., 2011; Scott et al., 2017; Zhao et al., 2017). These prompt further characterization of HLA variants and their relationship with melioidosis progression when modulated by patient diabetic status. However, genetic studies on HLA and melioidosis have suffered from small sample size and availability of reliable HLA typing platform. This is largely impeded by the under-representation of genomic data from the population from melioidosis endemic areas. The availability of data is crucial as this improves imputations and discovery of new causal variants and disease association.

## Discussion

The advancement in omic technologies has improved our understanding of co-evolution between *B. pseudomallei* and different hosts, thereby guiding better control policy, treatment option and vaccine design. Proteins or molecules that participate in host—*B. pseudomallei* interaction are extremely variable. Their variations have been seen at the genomic, epigenetic, transcriptomic and proteomic levels. Although genome data for human population from melioidois endemic areas is still scarce, genome data for *B.pseudomallei* has been accumulating (Holden et al., 2004; Hayden et al., 2012; Price et al., 2013, 2016; Sahl et al., 2013, 2016; Daligault et al., 2014; Bugrysheva et al., 2015; Chen et al., 2015; Hsueh et al., 2015; Johnson et al., 2015a,b; McRobb et al., 2015; Nandi et al., 2015; Sidjabat et al., 2015; Song et al., 2015; Spring-Pearson et al., 2015; Viberg et al., 2015; Chapple et al., 2016; Aziz et al., 2017; Chewapreecha et al., 2017, 2019; Podnecky et al., 2017; Viberg et al., 2017; Webb et al., 2019). When combined with spatial and temporal information, this allows further exploration of allelic variants and a shift in allele frequency over space and time. Moreover, a genome-wide saturation mutagenesis can aid prediction of essential genes required under certain conditions (Moule et al., 2015). This can be coupled with transcriptome information to understand variations in the expression patterns (Chieng et al., 2012; Ooi et al., 2013; Price et al., 2018) during the course of infection, and across different host types. A dual host-pathogen transcriptome study has not been conducted for melioidosis but is promising to provide valuable insight into the interaction as well as variations that lead to different infection outcomes.

In this article, we mainly explored variations of *B. pseudomallei* genes implicated in human host infections at the genetic level. Many of these genes display a strong geographical signal which could either be a result of a founder effect following a migration out of Australia, or an acquisition of new alleles required for local adaptation after an introduction to new geographical location. For each virulence gene, we also noted co-existence of multiple alleles in *B. pseudomallei* population isolated from the same geographical region. The genetic polymorphism could be maintained by balancing selection where each co-existing allele must be favored under different condition. In the context of virulent genes, these could involve competitions with different soil organisms. Signals for positive selection could be detected in genes that promote bacterial survival under hostile environment, and genes required for cell entry and adaptation to an intracellular lifestyle. The latter can be grouped as virulence genes although they may primarily be used in amoeba hosts rather than mammalian hosts. For multi-host—pathogen interaction, it is thus essential to consider virulence in a broad host and environmental context. The picture is far from complete at the moment, but more incoming omic data is promising to shed light on this complex relationship.

## Author Contributions

CCe conceived the study and performed data analyses. CCo and PB performed literature review. AS performed data analyses. All authors wrote the manuscript.

## Conflict of Interest

The authors declare that the research was conducted in the absence of any commercial or financial relationships that could be construed as a potential conflict of interest.

## Supplementary Material

Supplementary Text We estimated the proportion of infection outcome variations (survival vs. mortal outcomes after 28 days) explained by *B. pseudomallei* nucleotide polymorphisms (SNPs). SNPs were called from Chewapreecha et al. (2019, 2020) using kSNP v3 software. A filtering for minimum allele frequency at 0.01 (MAF = 0.01) resulted in 65,379 SNPs for the analysis. *B. pseudomallei* population was defined by PopPUNK (Lees et al., 2019). We employed the Genome-wide Complex Trait Analysis (GCTA) (Yang et al., 2011) to calculate *h*^2^ using genomic restricted maximum likelihood (GREML) while adjusting for population structure using population clusters defined by PopPUNK.

## References

[B1] Abbink F. C. Orendi J. M. de Beaufort A. J. (2001). Mother-to-child transmission of *Burkholderia pseudomallei*. *N. Engl. J. Med.* 344 1171–1172. 11302149 10.1056/NEJM200104123441516

[B2] Adams N. M. Grassmann S. Sun J. C. (2020). Clonal expansion of innate and adaptive lymphocytes. *Nat. Rev. Immunol.* 20 694–707. 10.1038/s41577-020-0307-4 32424244 PMC13119617

[B3] Akkaya M. Kwak K. Pierce S. K. (2020). B cell memory: builing two walls of protection against pathogens. *Nat. Rev. Immunol.* 20 229–238. 10.1038/s41577-019-0244-2 31836872 PMC7223087

[B4] Anuntagool N. Aramsri P. Panichakul T. Wuthiekanun V. R. Kinoshita R. White N. J. (2000). Antigenic heterogeneity of lipopolysaccharide among *Burkholderia pseudomallei* clinical isolates. *Southeast Asian J. Trop. Med. Public Health* 31(Suppl. 1) 146–152. 11414445

[B5] Anuntagool N. Wuthiekanun V. White N. J. Currie B. J. Sermswan R. W. Wongratanacheewin S. (2006). Lipopolysaccharide heterogeneity among *Burkholderia pseudomallei* from different geographic and clinical origins. *Am. J. Trop. Med. Hyg.* 74 348–352. 10.4269/ajtmh.2006.74.348 16525090

[B6] Atkins T. Prior R. Mack K. Russell P. Nelson M. Prior J. (2002). Characterisation of an acapsular mutant of *Burkholderia pseudomallei* identified by signature tagged mutagenesis. *J. Med. Microbiol.* 51 539–553. 10.1099/0022-1317-51-7-539 12132769

[B7] AuCoin D. P. Reed D. E. Marlenee N. L. Bowen R. A. Thorkildson P. Judy B. M. (2012). Polysaccharide specific monoclonal antibodies provide passive protection against intranasal challenge with *Burkholderia pseudomallei*. *PLoS One* 7:e35386. 10.1371/journal.pone.0035386 22530013 PMC3328442

[B8] Aziz A. Currie B. J. Mayo M. Sarovich D. S. Price E. P. (2020). Comparative genomics confirms a rare melioidosis human-to-human transmission event and reveals incorrect phylogenomic reconstruction due to polyclonality. *Microb. Genom.* 6:e000326. 10.1099/mgen.0.000326 PMC7067207 31958055

[B9] Aziz A. Sarovich D. S. Harris T. M. Kaestli M. McRobb E. Mayo M. (2017). Suspected cases of intracontinental *Burkholderia pseudomallei* sequence type homoplasy resolved using whole-genome sequencing. *Microb. Genom.* 3:e000139. 10.1099/mgen.0.000139 29208140 PMC5729916

[B10] Balder R. Lipski S. Lazarus J. J. Grose W. Wooten R. M. Hogan R. J. (2010). Identification of *Burkholderia mallei* and *Burkholderia pseudomallei* adhesins for human respiratory epithelial cells. *BMC Microbiol.* 10:250. 10.1186/1471-2180-10-250 20920184 PMC2955633

[B11] Benanti E. L. Nguyen C. M. Welch M. D. (2015). Virulent *Burkholderia* species mimic host actin polymerases to drive actin-based motility. *Cell* 161 348–360. 10.1016/j.cell.2015.02.044 25860613 PMC4393530

[B12] Birnie E. Weehuizen T. A. F. Lankelma J. M. de Jong H. K. Koh G. C. K. W. van Lieshout M. H. P. (2019). Role of toll-like receptor 5 (TLR5) in experimental melioidosis. *Infect. Immun.* 87 e409–e418. 10.1128/IAI.00409-18 PMC6652761 31109950

[B13] Bot A. Benites J. (2005). *The Importance of Soil Organic Matter: key to Drought-Resistant Soil and Sustained Food Production.* Rome: Food and Agriculture Organization of the United Nations, 78.

[B14] Breitbach K. Klocke S. Tschernig T. van Rooijen N. Baumann U. Steinmetz I. (2006). Role of inducible nitric oxide synthase and NADPH oxidase in early control of *Burkholderia pseudomallei* infection in mice. *Infect. Immun.* 74 6300–6309. 10.1128/iai.00966-06 17000727 PMC1695503

[B15] Brett P. J. Burtnick M. N. Heiss C. Azadi P. DeShazer D. Woods D. E. (2011). *Burkholderia thailandensis* oacA mutants facilitate the expression of *Burkholderia mallei*-like O polysaccharides. *Infect. Immun.* 79 961–969. 10.1128/iai.01023-10 21115721 PMC3028842

[B16] Brett P. J. Burtnick M. N. Woods D. E. (2003). The wbiA locus is required for the 2-O-acetylation of lipopolysaccharides expressed by *Burkholderia pseudomallei* and *Burkholderia thailandensis*. *FEMS Microbiol. Lett.* 218 323–328. 12586411 10.1111/j.1574-6968.2003.tb11536.x

[B17] Brett P. J. Mah D. C. Woods D. E. (1994). Isolation and characterization of *Pseudomonas pseudomallei* flagellin proteins. *Infect Immun.* 62 1914–1919. 10.1128/iai.62.5.1914-1919.1994 7513308 PMC186440

[B18] Brett P. J. Woods D. E. (1996). Structural and immunological characterization of *Burkholderia pseudomallei* O-polysaccharide-flagellin protein conjugates. *Infect. Immun.* 64 2824–2828. 10.1128/iai.64.7.2824-2828.1996 8698517 PMC174148

[B19] Brock D. A. Read S. Bozhchenko A. Queller D. C. Strassmann J. E. (2013). Social amoeba farmers carry defensive symbionts to protect and privatize their crops. *Nat. Commun.* 4:2385. 10.1038/ncomms3385 24029835

[B20] Broek C. W. V. Chalmers K. J. Stevens M. P. Stevens J. M. (2015). Quantitative proteomic analysis of *Burkholderia pseudomallei* Bsa type III secretion system effectors using hypersecreting mutants. *Mol. Cell. Proteom.* 14 905–916. 10.1074/mcp.m114.044875 25635268 PMC4390269

[B21] Brown N. F. Logue C. A. Boddey J. A. Scott R. Hirst R. G. Beacham I. R. (2004). Identification of a novel two-partner secretion system from *Burkholderia pseudomallei*. *Mol. Genet. Genom.* 272 204–215. 10.1007/s00438-004-1039-z 15316770

[B22] Bugrysheva J. V. Sue D. Hakovirta J. Loparev V. N. Knipe K. Sammons S. A. (2015). Finished Annotated Genome Sequence of *Burkholderia pseudomallei* Strain Bp1651, a Multidrug-Resistant Clinical Isolate. *Genome Announc.* 3:e01427-15. 10.1128/genomeA.01427-15 PMC4669406 26634765

[B23] Burtnick M. N. Brett P. J. Harding S. V. Ngugi S. A. Ribot W. J. Chantratita N. (2011). The Cluster 1 type VI secretion system is a major virulence determinant in *Burkholderia pseudomallei*. *Infect. Immun.* 79 1512–1525. 10.1128/iai.01218-10 21300775 PMC3067527

[B24] Campos C. G. Byrd M. S. Cotter P. A. (2013). Functional characterization of *Burkholderia pseudomallei* trimeric autotransporters. *Infect. Immun.* 81 2788–2799. 10.1128/iai.00526-13 23716608 PMC3719557

[B25] Capelli R. Peri C. Villa R. Nithichanon A. Conchillo-Solé O. Yero D. (2018). BPSL1626: reverse and structural vaccinology reveal a novel candidate for vaccine design against *Burkholderia pseudomallei*. *Antibodies (Basel)* 7:26. 10.3390/antib7030026 31544878 PMC6640674

[B26] Ceballos-Olvera I. Sahoo M. Miller M. A. Del Barrio L. Re F. (2011). Inflammasome-dependent pyroptosis and IL-18 protect against *Burkholderia pseudomallei* lung infection while IL-1β is deleterious. *PLoS Pathog.* 7:e1002452. 10.1371/journal.ppat.1002452 22241982 PMC3248555

[B27] Chaichana P. Chantratita N. Brod F. Koosakulnirand S. Jenjaroen K. Chumseng S. (2017). A nonsense mutation in TLR5 is associated with survival and reduced IL-10 and TNF-alpha levels in human melioidosis. *PLoS Negl. Trop. Dis.* 11:e0005587. 10.1371/journal.pntd.0005587 28475641 PMC5435357

[B28] Challacombe J. F. Stubben C. J. Klimko C. P. Welkos S. L. Kern S. J. Bozue J. A. (2014). Interrogation of the *Burkholderia pseudomallei* genome to address differential virulence among isolates. *PLoS One* 9:e115951. 10.1371/journal.pone.0115951 25536074 PMC4275268

[B29] Chantratita N. Tandhavanant S. Myers N. D. Chierakul W. Robertson J. D. Mahavanakul W. (2014). Screen of whole blood responses to flagellin identifies TLR5 variation associated with outcome in melioidosis. *Genes Immun.* 15 63–71. 10.1038/gene.2013.60 24285178 PMC3948086

[B30] Chapple S. N. J. Sarovich D. S. Holden M. T. G. Peacock S. J. Buller N. Golledge C. (2016). Whole-genome sequencing of a quarter-century melioidosis outbreak in temperate Australia uncovers a region of low-prevalence endemicity. *Microb. Genom.* 2:e000067. 10.1099/mgen.0.000067 PMC5343139 28348862

[B31] Chen Y. Schröder I. French C. T. Jaroszewicz A. Yee X. J. Teh B.-E. (2014). Characterization and analysis of the *Burkholderia pseudomallei* BsaN virulence regulon. *BMC Microbiol.* 14:206. 10.1186/s12866-014-0206-6 25085508 PMC4236580

[B32] Chen Y. Wong J. Sun G. W. Liu Y. Tan G.-Y. G. Gan Y.-H. (2011). Regulation of type VI secretion system during *Burkholderia pseudomallei* infection. *Infect. Immun.* 79 3064–3073. 10.1128/iai.05148-11 21670170 PMC3147588

[B33] Chen Y. L. Chen Y. S. Lin H. H. Chan C. W. Chen S. C. Chen C. H. (2007). Immunostimulatory flagellin from *Burkholderia pseudomallei* effects on an increase in the intracellular calcium concentration and up-regulation of TNF-alpha by mononuclear cells. *Microbiol. Immunol.* 51 81–86. 10.1111/j.1348-0421.2007.tb03893.x 17237602

[B34] Chen Y.-S. Lin H.-H. Hsueh P.-T. Liu P.-J. Ni W.-F. Chung W.-C. (2015). Whole-genome sequence of an epidemic strain of *Burkholderia pseudomallei* vgh07 in Taiwan. *Genome Announc.* 3:e00345-15. 10.1128/genomeA.00345-15 PMC4417695 25931599

[B35] Chewapreecha C. Harris S. R. Croucher N. J. Turner C. Marttinen P. Cheng L. (2014). Dense genomic sampling identifies highways of pneumococcal recombination. *Nat. Genet.* 46 305–309. 10.1038/ng.2895 24509479 PMC3970364

[B36] Chewapreecha C. Holden M. T. G. Vehkala M. Välimäki N. Yang Z. Harris S. R. (2017). Global and regional dissemination and evolution of *Burkholderia pseudomallei*. *Nat. Microbiol.* 2 1–8. 10.1038/nmicrobiol.2016.263 PMC5300093 28112723

[B37] Chewapreecha C. Mather A. E. Harris S. R. Hunt M. Holden M. T. G. Chaichana C. (2019). Genetic variation associated with infection and the environment in the accidental pathogen *Burkholderia pseudomallei*. *Commun. Biol.* 2 1–11. 31799430 10.1038/s42003-019-0678-x PMC6874650

[B38] Chewapreecha C. Pensar J. Chattagul S. Pesonen M. Sangphukieo A. Boonklang P. (2020). Co-evolutionary signals from *Burkholderia pseudomallei* population genomics highlight its survival strategy in a hostile environment. *bioRxiv* [Preprint] 10.1101/2020.08.11.245894

[B39] Chieng S. Carreto L. Nathan S. (2012). *Burkholderia pseudomallei* transcriptional adaptation in macrophages. *BMC Genom.* 13:328. 10.1186/1471-2164-13-328 22823543 PMC3418162

[B40] Chua K. L. Chan Y. Y. Gan Y. H. (2003). Flagella are virulence determinants of *Burkholderia pseudomallei*. *Infect. Immun.* 71 1622–1629. 10.1128/iai.71.4.1622-1629.2003 12654773 PMC152022

[B41] Chuaygud T. Tungpradabkul S. Sirisinha S. Chua K. L. Utaisincharoen P. (2008). A role of *Burkholderia pseudomallei* flagella as a virulent factor. *Trans. R. Soc. Trop. Med. Hyg.* 102(Suppl. 1) S140–S144. 19121676 10.1016/S0035-9203(08)70031-2

[B42] Clarke E. T. Williams N. A. Findlow J. Borrow R. Heyderman R. S. Finn A. (2013). Polysaccharide-specific memory B cells generated by conjugate vaccines in humans conform to the CD27+IgG+ isotype-switched memory B Cell phenotype and require contact-dependent signals from bystander T cells activated by bacterial proteins to differentiate into plasma cells. *J. Immunol.* 191 6071–6083. 10.4049/jimmunol.1203254 24227777

[B43] Cloutier M. Muru K. Ravicoularamin G. Gauthier C. (2018). Polysaccharides from *Burkholderia* species as targets for vaccine development, immunomodulation and chemical synthesis. *Nat. Prod. Rep.* 35 1251–1293. 10.1039/c8np00046h 30023998

[B44] Cooke G. S. Hill A. V. (2001). Genetics of susceptibility to human infectious disease. *Nat. Rev. Genet.* 2 967–977. 11733749 10.1038/35103577

[B45] Cuccui J. Milne T. S. Harmer N. George A. J. Harding S. V. Dean R. E. (2012). Characterization of the *Burkholderia pseudomallei* K96243 capsular polysaccharide I coding region. *Infect. Immun.* 80 1209–1221. 10.1128/iai.05805-11 22252864 PMC3294636

[B46] Currie B. J. Jacups S. P. (2003). Intensity of rainfall and severity of melioidosis, Australia. *Emerg. Infect. Dis. J. CDC* 9 1538–1542. 10.3201/eid0912.020750 14720392 PMC3034332

[B47] Daligault H. E. Davenport K. W. Minogue T. D. Bishop-Lilly K. A. Broomall S. M. Bruce D. C. (2014). Whole-genome assemblies of 56 *Burkholderia* species. *Genome Announc.* 2:e01106-14. 10.1128/genomeA.01106-14 PMC4239345 25414490

[B48] David S. Sanchez-Buso L. Harris S. R. Marttinen P. Rusniok C. Buchrieser C. (2017). Dynamics and impact of homologous recombination on the evolution of *Legionella pneumophila*. *PLoS Genet.* 13:e1006855. 10.1371/journal.pgen.1006855 28650958 PMC5507463

[B49] de Bakker P. I. W. McVean G. Sabeti P. C. Miretti M. M. Green T. Marchini J. (2006). A high-resolution HLA and SNP haplotype map for disease association studies in the extended human MHC. *Nat. Genet.* 38 1166–1172. 10.1038/ng1885 16998491 PMC2670196

[B50] Dendrou C. A. Petersen J. Rossjohn J. Fugger L. (2018). HLA variation and disease. *Nat. Rev. Immunol.* 18 325–339. 29292391 10.1038/nri.2017.143

[B51] DeShazer D. Brett P. J. Carlyon R. Woods D. E. (1997). Mutagenesis *of Burkholderia pseudomallei* with Tn5-OT182: isolation of motility mutants and molecular characterization of the flagellin structural gene. *J. Bacteriol.* 179 2116–2125. 10.1128/jb.179.7.2116-2125.1997 9079894 PMC178945

[B52] Dharakul T. Vejbaesya S. Chaowagul W. Luangtrakool P. Stephens H. A. Songsivilai S. (1998). HLA-DR and -DQ associations with melioidosis. *Hum. Immunol.* 59 580–586. 10.1016/s0198-8859(98)00052-4 9757914

[B53] Dickey A. K. Chantratita N. Tandhavanant S. Ducken D. Lovelace-Macon L. Seal S. (2019). Flagellin-independent effects of a Toll-like receptor 5 polymorphism in the inflammatory response to *Burkholderia pseudomallei*. *PLoS Neg. Trop. Dis.* 13:e0007354. 10.1371/journal.pntd.0007354 31067234 PMC6527242

[B54] DiSalvo S. Haselkorn T. S. Bashir U. Jimenez D. Brock D. A. Queller D. C. (2015). *Burkholderia* bacteria infectiously induce the proto-farming symbiosis of Dictyostelium amoebae and food bacteria. *Proc. Natl. Acad. Sci. U.S.A.* 112 E5029–E5037. 26305954 10.1073/pnas.1511878112 PMC4568666

[B55] Dowling A. J. Wilkinson P. A. Holden M. T. G. Quail M. A. Bentley S. D. Reger J. (2010). Genome-wide analysis reveals loci encoding anti-macrophage factors in the human pathogen *Burkholderia pseudomallei* K96243. *PLoS One* 5:e15693. 10.1371/journal.pone.0015693 21203527 PMC3008741

[B56] Duangsonk K. Gal D. Mayo M. Hart C. A. Currie B. J. Winstanley C. (2006). Use of a variable amplicon typing scheme reveals considerable variation in the accessory genomes of isolates of *Burkholderia pseudomallei*. *J. Clin. Microbiol.* 44 1323–1334. 10.1128/jcm.44.4.1323-1334.2006 16597858 PMC1448637

[B57] Dunachie S. J. Jenjaroen K. Reynolds C. J. Quigley K. J. Sergeant R. Sumonwiriya M. (2017). Infection with *Burkholderia pseudomallei* – immune correlates of survival in acute melioidosis. *Sci. Rep.* 7:12143. 10.1038/s41598-017-12331-5 PMC5610189 28939855

[B58] Easton A. Haque A. Chu K. Lukaszewski R. Bancroft G. J. A. (2007). Critical role for neutrophils in resistance to experimental infection with *Burkholderia pseudomallei*. *J. Infect. Dis.* 195 99–107. 10.1086/509810 17152013

[B59] Ewbank D. (2016). Measuring selection in human populations using the growth rate per generation. *Philos. Trans. R. Soc. Lond. B Biol. Sci.* 371:20150148. 10.1098/rstb.2015.0148 27022075 PMC4822428

[B60] Felgner P. L. Kayala M. A. Vigil A. Burk C. Nakajima-Sasaki R. Pablo J. (2009). A *Burkholderia pseudomallei* protein microarray reveals serodiagnostic and cross-reactive antigens. *Proc. Natl. Acad. Sci. U.S.A.* 106 13499–13504. 10.1073/pnas.0812080106 19666533 PMC2717108

[B61] Finucane H. K. Bulik-Sullivan B. Gusev A. Trynka G. Reshef Y. Loh P. R. (2015). Partitioning heritability by functional annotation using genome-wide association summary statistics. *Nat. Genet.* 47 1228–1235. 10.1038/ng.3404 26414678 PMC4626285

[B62] French C. T. Toesca I. J. Wu T.-H. Teslaa T. Beaty S. M. Wong W. (2011). Dissection of the *Burkholderia* intracellular life cycle using a photothermal nanoblade. *Proc.Natl. Acad. Sci. U.S.A.* 108 12095–12100. 10.1073/pnas.1107183108 21730143 PMC3141958

[B63] Gourraud P.-A. Khankhanian P. Cereb N. Yang S. Y. Feolo M. Maiers M. (2014). HLA diversity in the 1000 genomes dataset. *PLoS One* 9:e97282. 10.1371/journal.pone.0097282 24988075 PMC4079705

[B64] Gregory A. E. Judy B. M. Qazi O. Blumentritt C. A. Brown K. A. Shaw A. M. (2015). A gold nanoparticle-linked glycoconjugate vaccine against *Burkholderia mallei*. *Nanomedicine* 11 447–456. 10.1016/j.nano.2014.08.005 25194998 PMC4330121

[B65] Gutierrez M. G. Warawa J. M. (2016). Attenuation of a select agent-excluded *Burkholderia pseudomallei* capsule mutant in hamsters. *Acta Trop.* 157 68–72. 10.1016/j.actatropica.2015.12.006 26836271

[B66] Hantrakun V. Rongkard P. Oyuchua M. Amornchai P. Lim C. Wuthiekanun V. (2016). Soil nutrient depletion is associated with the presence of *Burkholderia pseudomallei*. *Appl. Environ. Microbiol.* 82 7086–7092. 27694236 10.1128/AEM.02538-16 PMC5118919

[B67] Haselkorn T. S. DiSalvo S. Miller J. W. Bashir U. Brock D. A. Queller D. C. (2019). The specificity of *Burkholderia* symbionts in the social amoeba farming symbiosis: prevalence, species, genetic and phenotypic diversity. *Mol. Ecol.* 28 847–862. 10.1111/mec.14982 30575161

[B68] Hayashi F. Smith K. D. Ozinsky A. Hawn T. R. Yi E. C. Goodlett D. R. (2001). The innate immune response to bacterial flagellin is mediated by Toll-like receptor 5. *Nature* 410 1099–1103. 10.1038/35074106 11323673

[B69] Hayden H. S. Lim R. Brittnacher M. J. Sims E. H. Ramage E. R. Fong C. (2012). Evolution of *Burkholderia pseudomallei* in recurrent melioidosis. *PLoS One.* 7:e36507. 10.1371/journal.pone.0036507 22615773 PMC3352902

[B70] Heiss C. Burtnick M. N. Roberts R. A. Black I. Azadi P. Brett P. J. (2013). Revised structures for the predominant O-polysaccharides expressed by *Burkholderia pseudomallei* and *Burkholderia mallei*. *Carbohydr. Res.* 381 6–11. 10.1016/j.carres.2013.08.013 24056008 PMC3923507

[B71] Holden M. T. G. Titball R. W. Peacock S. J. Cerdeño-Tárraga A. M. Atkins T. Crossman L. C. (2004). Genomic plasticity of the causative agent of melioidosis, *Burkholderia pseudomallei*. *Proc. Natl. Acad. Sci. U.S.A.* 101 14240–14245. 15377794 10.1073/pnas.0403302101 PMC521101

[B72] Hou K. Burch K. S. Majumdar A. Shi H. Mancuso N. Wu Y. (2019). Accurate estimation of SNP-heritability from biobank-scale data irrespective of genetic architecture. *Nat. Genet.* 51 1244–1251. 10.1038/s41588-019-0465-0 31358995 PMC6686906

[B73] Hsueh P.-T. Chen Y.-S. Lin H.-H. Liu P.-J. Ni W.-F. Liu M.-C. (2015). Comparison of whole-genome sequences from two colony morphovars of *Burkholderia pseudomallei*. *Genome Announc.* 3:e01194-15. 10.1128/genomeA.01194-15 PMC4611688 26472836

[B74] Hu D. Reeves P. R. (2020). The remarkable dual-level diversity of prokaryotic flagellins. *mSystems* 5:e00705-19. 10.1128/mSystems.00705-19 PMC7018530 32047063

[B75] Inglis T. J. Rigby P. Robertson T. A. Dutton N. S. Henderson M. Chang B. J. (2000). Interaction between *Burkholderia pseudomallei* and *Acanthamoeba* species results in coiling phagocytosis, endamebic bacterial survival, and escape. *Infect. Immun.* 68 1681–1686. 10.1128/iai.68.3.1681-1686.2000 10678988 PMC97329

[B76] Inglis T. J. Robertson T. Woods D. E. Dutton N. Chang B. J. (2003). Flagellum-mediated adhesion by *Burkholderia pseudomallei* precedes invasion of *Acanthamoeba astronyxis*. *Infect. Immun.* 71 2280–2282. 10.1128/iai.71.4.2280-2282.2003 12654857 PMC152052

[B77] Jenjaroen K. Chumseng S. Sumonwiriya M. Ariyaprasert P. Chantratita N. Sunyakumthorn P. (2015). T-Cell responses are associated with survival in acute melioidosis patients. *PLoS Neg. Trop. Dis.* 9:e0004152. 10.1371/journal.pntd.0004152 26495852 PMC4619742

[B78] Jia G. Li Y. Zhang H. Chattopadhyay I. Boeck Jensen A. Blair D. R. (2019). Estimating heritability and genetic correlations from large health datasets in the absence of genetic data. *Nat. Commun.* 10:5508. 10.1038/s41467-019-13455-0 PMC6890770 31796735

[B79] Johnson S. L. Baker A. L. Chain P. S. Currie B. J. Daligault H. E. Davenport K. W. (2015a). Whole-Genome Sequences of 80 Environmental and Clinical Isolates of *Burkholderia pseudomallei*. *Genome Announc.* 3:e01282-14. 10.1128/genomeA.01282-14 PMC4333647 25676747

[B80] Johnson S. L. Bishop-Lilly K. A. Ladner J. T. Daligault H. E. Davenport K. W. Jaissle J. (2015b). Complete genome sequences for 59 *burkholderia* isolates, both pathogenic and near neighbor. *Genome Announc.* 3:e00159-15. 10.1128/genomeA.00159-15 PMC4417688 25931592

[B81] Kariuki S. N. Marin-Menendez A. Introini V. Ravenhill B. J. Lin Y. C. Macharia A. (2020). Red blood cell tension protects against severe malaria in the Dantu blood group. *Nature.* 585 579–583. 10.1038/s41586-020-2726-6 32939086 PMC7116803

[B82] Karlsson E. K. Kwiatkowski D. P. Sabeti P. C. (2014). Natural selection and infectious disease in human populations. *Nat. Rev. Genet.* 15 379–393. 10.1038/nrg3734 24776769 PMC4912034

[B83] Khojandi N. Haselkorn T. S. Eschbach M. N. Naser R. A. DiSalvo S. (2019). Intracellular *Burkholderia* Symbionts induce extracellular secondary infections; driving diverse host outcomes that vary by genotype and environment. *ISME J.* 13 2068–2081. 10.1038/s41396-019-0419-7 31019270 PMC6776111

[B84] King J. D. Berry S. Clarke B. R. Morris R. J. Whitfield C. (2014). Lipopolysaccharide O antigen size distribution is determined by a chain extension complex of variable stoichiometry in *Escherichia coli* O9a. *Proc. Natl. Acad. Sci. U.S.A.* 111 6407–6412. 10.1073/pnas.1400814111 24733938 PMC4035927

[B85] Knirel Y. A. Paramonov N. A. Shashkov A. S. Kochetkov N. K. Yarullin R. G. Farber S. M. (1992). Structure of the polysaccharide chains of *Pseudomonas pseudomallei* lipopolysaccharides. *Carbohydr. Res.* 233 185–193. 10.1016/s0008-6215(00)90930-3 1280183

[B86] Koonpaew S. Ubol M. N. Sirisinha S. White N. J. Chaiyaroj S. C. (2000). Genome fingerprinting by pulsed-field gel electrophoresis of isolates of *Burkholderia pseudomallei* from patients with melioidosis in Thailand. *Acta Trop.* 74 187–191. 10.1016/s0001-706x(99)00069-8 10674648

[B87] Koosakulnirand S. Phokrai P. Jenjaroen K. Roberts R. A. Utaisincharoen P. Dunachie S. J. (2018). Immune response to recombinant *Burkholderia pseudomallei* FliC. *PLoS One* 13:e0198906. 10.1371/journal.pone.0198906 29902230 PMC6002054

[B88] Kronsteiner B. Chaichana P. Sumonwiriya M. Jenjaroen K. Chowdhury F. R. Chumseng S. (2019). Diabetes alters immune response patterns to acute melioidosis in humans. *Eur. J. Immunol.* 49 1092–1106. 10.1002/eji.201848037 31032897 PMC6618312

[B89] Lazar Adler N. Stevens J. Stevens M. Galyov E. (2011). Autotransporters and their role in the virulence of *Burkholderia pseudomallei* and *Burkholderia mallei*. *Front. Microbiol.* 2:151. 10.3389/fmicb.2011.00151 21811486 PMC3139927

[B90] Lazar Adler N. R. Stevens M. P. Dean R. E. Saint R. J. Pankhania D. Prior J. L. (2015). Systematic mutagenesis of genes encoding predicted autotransported proteins of *Burkholderia pseudomallei* identifies factors mediating virulence in mice, net intracellular replication and a novel protein conferring serum resistance. *PLoS One* 10:e0121271. 10.1371/journal.pone.0121271 25830295 PMC4382181

[B91] Lees J. A. Croucher N. J. Goldblatt D. Nosten F. Parkhill J. Turner C. (2017). Genome-wide identification of lineage and locus specific variation associated with pneumococcal carriage duration. *Elife* 6:e26255. 10.7554/eLife.26255 PMC5576492 28742023

[B92] Lees J. A. Harris S. R. Tonkin-Hill G. Gladstone R. A. Lo S. W. Weiser J. N. (2019). Fast and flexible bacterial genomic epidemiology with PopPUNK. *Genome Res.* 29 304–316. 10.1101/gr.241455.118 30679308 PMC6360808

[B93] Lennings J. West T. E. Schwarz S. (2019). The *Burkholderia* type VI secretion system 5: composition, regulation and role in virulence. *Front. Microbiol.* 9:3339. 10.3389/fmicb.2018.03339 30687298 PMC6335564

[B94] Lieberman T. D. Flett K. B. Yelin I. Martin T. R. McAdam A. J. Priebe G. P. (2014). Genetic variation of a bacterial pathogen within individuals with cystic fibrosis provides a record of selective pressures. *Nat. Genet.* 46 82–87. 10.1038/ng.2848 24316980 PMC3979468

[B95] Limmathurotsakul D. Holden M. T. G. Coupland P. Price E. P. Chantratita N. Wuthiekanun V. (2014a). Microevolution of *Burkholderia pseudomallei* during an acute infection. *J. Clin. Microbiol.* 52 3418–3421. 10.1128/jcm.01219-14 24966357 PMC4313173

[B96] Limmathurotsakul D. Paeyao A. Wongratanacheewin S. Saiprom N. Takpho N. Thaipadungpanit J. (2014b). Role of *Burkholderia pseudomallei* biofilm formation and lipopolysaccharide in relapse of melioidosis. *Clin. Microbiol. Infect.* 20 O854–O856. 24602145 10.1111/1469-0691.12614 PMC4304327

[B97] Limmathurotsakul D. Wongratanacheewin S. Teerawattanasook N. Wongsuvan G. Chaisuksant S. Chetchotisakd P. (2010a). Increasing incidence of human melioidosis in Northeast Thailand. *Am. J. Trop. Med. Hyg.* 82 1113–1117. 10.4269/ajtmh.2010.10-0038 20519609 PMC2877420

[B98] Limmathurotsakul D. Wuthiekanun V. Chantratita N. Wongsuvan G. Amornchai P. Day N. P. J. (2010b). *Burkholderia pseudomallei* is spatially distributed in soil in Northeast Thailand. *PLoS Neg. Trop. Dis.* 4:e694. 10.1371/journal.pntd.0000694 20532233 PMC2879387

[B99] Losada L. Ronning C. M. DeShazer D. Woods D. Fedorova N. Kim H. S. (2010). Continuing evolution of *Burkholderia mallei* through genome reduction and large-scale rearrangements. *Genome Biol. Evol.* 2 102–116. 10.1093/gbe/evq003 20333227 PMC2839346

[B100] Mahasirimongkol S. Yanai H. Mushiroda T. Promphittayarat W. Wattanapokayakit S. Phromjai J. (2012). Genome-wide association studies of tuberculosis in Asians identify distinct at-risk locus for young tuberculosis. *J. Hum. Genet.* 57 363–367. 10.1038/jhg.2012.35 22551897

[B101] Malaria Genomic Epidemiology Network (2008). A global network for investigating the genomic epidemiology of malaria. *Nature* 456 732–737. 10.1038/nature07632 19079050 PMC3758999

[B102] Manivanh L. Pierret A. Rattanavong S. Kounnavongsa O. Buisson Y. Elliott I. (2017). *Burkholderia pseudomallei* in a lowland rice paddy: seasonal changes and influence of soil depth and physico-chemical properties. *Sci. Rep.* 7:3031. 10.1038/s41598-017-02946-z PMC5465195 28596557

[B103] McRobb E. Sarovich D. S. Price E. P. Kaestli M. Mayo M. Keim P. (2015). Tracing melioidosis back to the source: using whole-genome sequencing to investigate an outbreak originating from a contaminated domestic water supply. *J. Clin. Microbiol.* 53 1144–1148. 10.1128/jcm.03453-14 25631791 PMC4365233

[B104] Moore R. A. Tuanyok A. Woods D. E. (2008). Survival of *Burkholderia pseudomallei* in water. *BMC Res. Notes* 1:11. 10.1186/1756-0500-1-11 PMC2518269 18710531

[B105] Moule M. G. Spink N. Willcocks S. Lim J. Guerra-Assunção J. A. Cia F. (2015). Characterization of new virulence factors involved in the intracellular growth and survival of *Burkholderia pseudomallei*. *Infect. Immun.* 84 701–710. 10.1128/iai.01102-15 26712202 PMC4771355

[B106] Myers N. D. Chantratita N. Berrington W. R. Chierakul W. Limmathurotsakul D. Wuthiekanun V. (2014). The role of NOD2 in murine and human melioidosis. *J. Immunol.* 192 300–307. 10.4049/jimmunol.1301436 24298015 PMC3872087

[B107] Nandi T. Holden M. T. G. Didelot X. Mehershahi K. Boddey J. A. Beacham I. (2015). *Burkholderia pseudomallei* sequencing identifies genomic clades with distinct recombination, accessory, and epigenetic profiles. *Genome Res.* 25 129–141. 10.1101/gr.177543.114 25236617 PMC4317168

[B108] Nandi T. Ong C. Singh A. P. Boddey J. Atkins T. Sarkar-Tyson M. (2010). A genomic survey of positive selection in *Burkholderia pseudomallei* provides insights into the evolution of accidental virulence. *PLoS Pathog.* 6:e1000845. 10.1371/journal.ppat.1000845 20368977 PMC2848565

[B109] Nelson M. Prior J. L. Lever M. S. Jones H. E. Atkins T. P. Titball R. W. (2004). Evaluation of lipopolysaccharide and capsular polysaccharide as subunit vaccines against experimental melioidosis. *J. Med. Microbiol.* 53(Pt 12) 1177–1182. 10.1099/jmm.0.45766-0 15585494

[B110] Nikolich-Zugich J. Slifka M. K. Messaoudi I. (2004). The many important facets of T-cell repertoire diversity. *Nat. Rev. Immunol.* 4 123–132. 10.1038/nri1292 15040585

[B111] Noinarin P. Chareonsudjai P. Wangsomnuk P. Wongratanacheewin S. Chareonsudjai S. (2016). environmental free-living amoebae isolated from soil in khon kaen, Thailand, Antagonize *Burkholderia pseudomallei*. *PLoS One* 11:e0167355. 10.1371/journal.pone.0167355 27898739 PMC5127566

[B112] Norris M. H. Schweizer H. P. Tuanyok A. (2017). Structural diversity of *Burkholderia pseudomallei* lipopolysaccharides affects innate immune signaling. *PLoS Neg. Trop. Dis.* 11:e0005571. 10.1371/journal.pntd.0005571 28453531 PMC5425228

[B113] Novem V. Shui G. Wang D. Bendt A. K. Sim S. H. Liu Y. (2009). Structural and biological diversity of lipopolysaccharides from *Burkholderia pseudomallei* and *Burkholderia thailandensis*. *Clin. Vaccine Immunol.* 16 1420–1428. 10.1128/cvi.00472-08 19692625 PMC2756838

[B114] Ooi W. F. Ong C. Nandi T. Kreisberg J. F. Chua H. H. Sun G. (2013). The condition-dependent transcriptional landscape of *Burkholderia pseudomallei*. *PLoS Genet.* 9:e1003795. 10.1371/journal.pgen.1003795 24068961 PMC3772027

[B115] Ou K. Ong C. Koh S. Y. Rodrigues F. Sim S. H. Wong D. (2005). Integrative genomic, transcriptional, and proteomic diversity in natural isolates of the human pathogen *Burkholderia pseudomallei*. *J. Bacteriol.* 187 4276–4285. 10.1128/jb.187.12.4276-4285.2005 15937190 PMC1151743

[B116] Paksanont S. Sintiprungrat K. Yimthin T. Pumirat P. Peacock S. J. Chantratita N. (2018). Effect of temperature on *Burkholderia pseudomallei* growth, proteomic changes, motility and resistance to stress environments. *Sci. Rep.* 8:9167. 10.1038/s41598-018-27356-7 PMC6004011 29907803

[B117] Panina E. M. Mattoo S. Griffith N. Kozak N. A. Yuk M. H. Miller J. F. (2005). A genome-wide screen identifies a Bordetella type III secretion effector and candidate effectors in other species. *Mol. Microbiol.* 58 267–279. 10.1111/j.1365-2958.2005.04823.x 16164564

[B118] Park B. S. Lee J. O. (2013). Recognition of lipopolysaccharide pattern by TLR4 complexes. *Exp. Mol. Med.* 45:e66. 10.1038/emm.2013.97 24310172 PMC3880462

[B119] Park J. M. Ghosh S. O’Connor T. J. (2020). Combinatorial selection in amoebal hosts drives the evolution of the human pathogen *Legionella pneumophila*. *Nat. Microbiol.* 5 599–609. 10.1038/s41564-019-0663-7 31988381 PMC10234074

[B120] Parthasarathy N. DeShazer D. England M. Waag D. M. (2006). Polysaccharide microarray technology for the detection of *Burkholderia pseudomallei* and *Burkholderia mallei* antibodies. *Diagn. Microbiol. Infect. Dis.* 56 329–332. 10.1016/j.diagmicrobio.2006.04.018 16765554 PMC7127370

[B121] Pearson T. Giffard P. Beckstrom-Sternberg S. Auerbach R. Hornstra H. Tuanyok A. (2009). Phylogeographic reconstruction of a bacterial species with high levels of lateral gene transfer. *BMC Biol.* 7:78. 10.1186/1741-7007-7-78 19922616 PMC2784454

[B122] Pearson T. Sahl J. W. Hepp C. M. Handady K. Hornstra H. Vazquez A. J. (2020). Pathogen to commensal? Longitudinal within-host population dynamics, evolution, and adaptation during a chronic >16-year *Burkholderia pseudomallei* infection. *PLoS Pathog.* 16:e1008298. 10.1371/journal.ppat.1008298 32134991 PMC7077878

[B123] Perry M. B. MacLean L. L. Schollaardt T. Bryan L. E. Ho M. (1995). Structural characterization of the lipopolysaccharide O antigens of *Burkholderia pseudomallei*. *Infect. Immun.* 63 3348–3352. 10.1128/iai.63.9.3348-3352.1995 7543882 PMC173460

[B124] Pilatz S. Breitbach K. Hein N. Fehlhaber B. Schulze J. Brenneke B. (2006). Identification of *Burkholderia pseudomallei* genes required for the intracellular life cycle and in vivo virulence. *Infect. Immun.* 74 3576–3586. 10.1128/iai.01262-05 16714590 PMC1479254

[B125] Podnecky N. L. Rhodes K. A. Mima T. Drew H. R. Chirakul S. Wuthiekanun V. (2017). Mechanisms of resistance to folate pathway inhibitors in *Burkholderia pseudomallei* : deviation from the Norm. *mBio* 8:e01357-17. 10.1128/mBio.01357-17 PMC5587915 28874476

[B126] Pothlichet J. Quintana-Murci L. (2013). The genetics of innate immunity sensors and human disease. *Int. Rev. Immunol.* 32 157–208. 10.3109/08830185.2013.777064 23570315

[B127] Price E. P. Sarovich D. S. Mayo M. Tuanyok A. Drees K. P. Kaestli M. (2013). Within-host evolution of *Burkholderia pseudomallei* over a twelve-year chronic carriage infection. *mBio* 4:e00388-13. 10.1128/mBio.00388-13 PMC3735121 23860767

[B128] Price E. P. Sarovich D. S. Smith E. J. MacHunter B. Harrington G. Theobald V. (2016). Unprecedented melioidosis cases in Northern Australia caused by an asian *Burkholderia pseudomallei* strain identified by using large-scale comparative genomics. *Appl. Environ. Microbiol.* 82 954–963. 10.1128/aem.03013-15 26607593 PMC4725268

[B129] Price E. P. Viberg L. T. Kidd T. J. Bell S. C. Currie B. J. Sarovich D. S. (2018). Transcriptomic analysis of longitudinal *Burkholderia pseudomallei* infecting the cystic fibrosis lung. *Microb. Genom.* 4:e000194. 10.1099/mgen.0.000194 PMC6159556 29989529

[B130] Pumpuang A. Chantratita N. Wikraiphat C. Saiprom N. Day N. P. Peacock S. J. (2011). Survival of *Burkholderia pseudomallei* in distilled water for 16 years. *Trans. R. Soc. Trop. Med. Hyg.* 105 598–600. 10.1016/j.trstmh.2011.06.004 21764093 PMC3183224

[B131] Rachlin A. Mayo M. Webb J. R. Kleinecke M. Rigas V. Harrington G. (2020). Whole-genome sequencing of *Burkholderia pseudomallei* from an urban melioidosis hot spot reveals a fine-scale population structure and localised spatial clustering in the environment. *Sci. Rep.* 10:5443. 10.1038/s41598-020-62300-8 PMC7096523 32214186

[B132] Ralph A. McBride J. Currie B. J. (2004). Transmission of *Burkholderia pseudomallei* via breast milk in northern Australia. *Pediatr. Infect. Dis. J.* 23 1169–1171. 15626961

[B133] Reckseidler S. L. DeShazer D. Sokol P. A. Woods D. E. (2001). Detection of bacterial virulence genes by subtractive hybridization: identification of capsular polysaccharide of *Burkholderia pseudomallei* as a major virulence determinant. *Infect. Immun.* 69 34–44. 10.1128/iai.69.1.34-44.2001 11119486 PMC97852

[B134] Reckseidler-Zenteno S. L. DeVinney R. Woods D. E. (2005). The capsular polysaccharide of *Burkholderia pseudomallei* contributes to survival in serum by reducing complement factor C3b deposition. *Infect. Immun.* 73 1106–1115. 10.1128/iai.73.2.1106-1115.2005 15664954 PMC547107

[B135] Reckseidler-Zenteno S. L. Moore R. Woods D. E. (2009). Genetics and function of the capsules of *Burkholderia pseudomallei* and their potential as therapeutic targets. *Mini. Rev. Med. Chem.* 9 265–271. 10.2174/138955709787316047 19200030

[B136] Reckseidler-Zenteno S. L. Viteri D.-F. Moore R. Wong E. Tuanyok A. Woods D. E. (2010). Characterization of the type III capsular polysaccharide produced by *Burkholderia pseudomallei*. *J. Med. Microbiol.* 59(Pt 12) 1403–1414. 10.1099/jmm.0.022202-0 20724509

[B137] Robinson W. H. (2015). Sequencing the functional antibody repertoire–diagnostic and therapeutic discovery. *Nat. Rev. Rheumatol.* 11 171–182. 10.1038/nrrheum.2014.220 25536486 PMC4382308

[B138] Sahl J. W. Stone J. K. Gelhaus H. C. Warren R. L. Cruttwell C. J. Funnell S. G. (2013). Genome Sequence of Burkholderia pseudomallei NCTC 13392. *Genome Announc.* 1:e183-13. 10.1128/genomeA.00183-13 PMC3662813 23704173

[B139] Sahl J. W. Vazquez A. J. Hall C. M. Busch J. D. Tuanyok A. Mayo M. (2016). The effects of signal erosion and core genome reduction on the identification of diagnostic markers. *mBio* 7:9. 10.1128/mBio.00846-16 PMC5030356 27651357

[B140] Saiprom N. Sangsri T. Tandhavanant S. Sengyee S. Phunpang R. Preechanukul A. (2020). Genomic loss in environmental and isogenic morphotype isolates of *Burkholderia pseudomallei* is associated with intracellular survival and plaque-forming efficiency. *PLoS Neg. Trop. Dis.* 14:e0008590. 10.1371/journal.pntd.0008590 32991584 PMC7546507

[B141] Samatey F. A. Imada K. Nagashima S. Vonderviszt F. Kumasaka T. Yamamoto M. (2001). Structure of the bacterial flagellar protofilament and implications for a switch for supercoiling. *Nature* 410 331–337. 10.1038/35066504 11268201

[B142] Sanchez-Buso L. Comas I. Jorques G. Gonzalez-Candelas F. (2014). Recombination drives genome evolution in outbreak-related *Legionella pneumophila* isolates. *Nat. Genet.* 46 1205–1211. 10.1038/ng.3114 25282102

[B143] Sarkar-Tyson M. Thwaite J. E. Harding S. V. Smither S. J. Oyston P. C. F. Atkins T. P. (2007). Polysaccharides and virulence of *Burkholderia pseudomallei*. *J. Med. Microbiol.* 56(Pt 8) 1005–1010. 17644705 10.1099/jmm.0.47043-0

[B144] Sarovich D. S. Garin B. Smet B. D. Kaestli M. Mayo M. Vandamme P. (2016). Phylogenomic analysis reveals an Asian Origin for African *Burkholderia pseudomallei* and further supports melioidosis endemicity in Africa. *mSphere* 1:e00089-15. 10.1128/mSphere.00089-15 PMC4863585 27303718

[B145] Sarovich D. S. Price E. P. Webb J. R. Ward L. M. Voutsinos M. Y. Tuanyok A. (2014). Variable virulence factors in *Burkholderia pseudomallei* (Melioidosis) associated with human disease. *PLoS One* 9:e91682. 10.1371/journal.pone.0091682 24618705 PMC3950250

[B146] Schuppler M. (2014). How the interaction of Listeria monocytogenes and Acanthamoeba spp. affects growth and distribution of the food borne pathogen. *Appl. Microbiol. Biotechnol.* 98 2907–2916. 10.1007/s00253-014-5546-5 24557567

[B147] Scott A. E. Burtnick M. N. Stokes M. G. M. Whelan A. O. Williamson E. D. Atkins T. P. (2014b). *Burkholderia pseudomallei* capsular polysaccharide conjugates provide protection against acute melioidosis. *Infect. Immun.* 82 3206–3213. 10.1128/iai.01847-14 24866807 PMC4136211

[B148] Scott A. E. Ngugi S. A. Laws T. R. Corser D. Lonsdale C. L. D’Elia R. V. (2014a). Protection against experimental melioidosis following immunisation with a lipopolysaccharide-protein conjugate. *J. Immunol. Res.* 2014: 392170. 10.1155/2014/392170 PMC4033506 24892035

[B149] Scott R. A. Scott L. J. Mägi R. Marullo L. Gaulton K. J. Kaakinen M. (2017). An expanded genome-wide association study of type 2 diabetes in Europeans. *Diabetes* 66:2888. 10.2337/db16-1253 PMC5652602 28566273

[B150] Shaw T. Tellapragada C. Kamath A. Kalwaje Eshwara V. Mukhopadhyay C. (2019). Implications of environmental and pathogen-specific determinants on clinical presentations and disease outcome in melioidosis patients. *PLoS Neg. Trop. Dis.* 13:e0007312. 10.1371/journal.pntd.0007312 31091290 PMC6538188

[B151] Shimizu Y. Ao H. Soemantri A. Tiwawech D. Settheetham-Ishida W. Kayame O. W. (2000). Sero- and molecular typing of Duffy blood group in Southeast Asians and Oceanians. *Hum. Biol.* 72 511–518. 10885196

[B152] Sidjabat H. E. Cottrell K. Cervin A. (2015). Draft genome sequences of *Burkholderia pseudomallei* and *Staphylococcus aureus*, Isolated from a Patient with Chronic Rhinosinusitis. *Genome Announc.* 3:e01075-15. 10.1128/genomeA.01075-15 PMC4591299 26430027

[B153] Silverman J. M. Brunet Y. R. Cascales E. Mougous J. D. (2012). Structure and regulation of the type VI secretion system. *Annu. Rev. Microbiol.* 66 453–472. 10.1146/annurev-micro-121809-151619 22746332 PMC3595004

[B154] Sim S. H. Yu Y. Lin C. H. Karuturi R. K. M. Wuthiekanun V. Tuanyok A. (2008). The core and accessory genomes of *Burkholderia pseudomallei*: implications for human melioidosis. *PLoS Pathog.* 4:e1000178. 10.1371/journal.ppat.1000178 18927621 PMC2564834

[B155] Sironi M. Cagliani R. Forni D. Clerici M. (2015). Evolutionary insights into host–pathogen interactions from mammalian sequence data. *Nat. Rev. Genet.* 16 224–236. 10.1038/nrg3905 25783448 PMC7096838

[B156] Sitthidet C. Stevens J. M. Chantratita N. Currie B. J. Peacock S. J. Korbsrisate S. (2008). Prevalence and sequence diversity of a factor required for actin-based motility in natural populations of *Burkholderia* species. *J. Clin. Microbiol.* 46 2418–2422. 10.1128/jcm.00368-08 18495853 PMC2446894

[B157] Slodkowicz G. Goldman N. (2020). Integrated structural and evolutionary analysis reveals common mechanisms underlying adaptive evolution in mammals. *Proc. Natl. Acad. Sci. U.S.A.* 117 5977–5986. 10.1073/pnas.1916786117 32123117 PMC7084095

[B158] Song L. Yu Y. Feng L. He J. Wang T. Zhu H. (2015). Draft genome sequence of *Burkholderia pseudomallei* strain 350105, isolated in Hainan, China, in 1976. *Genome Announc.* 3:e01162-15. 10.1128/genomeA.01162-15 PMC4611679 26472827

[B159] Southeast Asia Infectious Disease Clinical Research Network (2017). Causes and outcomes of sepsis in southeast Asia: a multinational multicentre cross-sectional study. *Lancet Glob. Health* 5 e157–e167. 28104185 10.1016/S2214-109X(17)30007-4 PMC5332551

[B160] Speed D. Holmes J. Balding D. J. (2020). Evaluating and improving heritability models using summary statistics. *Nat. Genet.* 52 458–462. 10.1038/s41588-020-0600-y 32203469

[B161] Spring-Pearson S. M. Stone J. K. Doyle A. Allender C. J. Okinaka R. T. Mayo M. (2015). Pangenome analysis of *Burkholderia pseudomallei*: genome evolution preserves gene order despite high recombination rates. *PLoS One* 10:e0140274. 10.1371/journal.pone.0140274 26484663 PMC4613141

[B162] Stevens J. M. Ulrich R. L. Taylor L. A. Wood M. W. DeShazer D. Stevens M. P. (2005). Actin-Binding proteins from *Burkholderia mallei* and *Burkholderia thailandensis* can functionally compensate for the actin-based motility defect of a *Burkholderia pseudomallei* bimA Mutant. *J. Bacteriol.* 187 7857–7862. 10.1128/jb.187.22.7857-7862.2005 16267310 PMC1280302

[B163] Stevens M. P. Stevens J. M. Jeng R. L. Taylor L. A. Wood M. W. Hawes P. (2005). Identification of a bacterial factor required for actin-based motility of *Burkholderia pseudomallei*. *Mol. Microbiol.* 56 40–53. 10.1111/j.1365-2958.2004.04528.x 15773977

[B164] Stone J. K. Mayo M. Grasso S. A. Ginther J. L. Warrington S. D. Allender C. J. (2012). Detection of *Burkholderia pseudomallei* O-antigen serotypes in near-neighbor species. *BMC Microbiol.* 12:250. 10.1186/1471-2180-12-250 23126230 PMC3541218

[B165] Suwannasaen D. Mahawantung J. Chaowagul W. Limmathurotsakul D. Felgner P. L. Davies H. (2011). Human immune responses to *Burkholderia pseudomallei* characterized by protein microarray analysis. *J. Infect. Dis.* 203 1002–1011. 10.1093/infdis/jiq142 21300673 PMC3068035

[B166] Sweeney T. E. Perumal T. M. Henao R. Nichols M. Howrylak J. A. Choi A. M. (2018). A community approach to mortality prediction in sepsis via gene expression analysis. *Nat. Commun.* 9:694. 10.1038/s41467-018-03078-2 PMC5814463 29449546

[B167] Tay S. T. Cheah P. C. Puthucheary S. D. (2010). Sequence polymorphism and PCR-restriction fragment length polymorphism analysis of the flagellin gene of *Burkholderia pseudomallei*. *J. Clin. Microbiol.* 48 1465–1467. 10.1128/jcm.01131-09 20089759 PMC2849561

[B168] Thomas A. D. Forbes-Faulkner J. Parker M. (1979). Isolation of *Pseudomonas pseudomallei* from clay layers at defined depths. *Am. J. Epidemiol.* 110 515–521. 10.1093/oxfordjournals.aje.a112832 507042

[B169] Thye T. Owusu-Dabo E. Vannberg F. O. van Crevel R. Curtis J. Sahiratmadja E. (2012). Common variants at 11p13 are associated with susceptibility to tuberculosis. *Nat. Genet.* 44 257–259. 10.1038/ng.1080 22306650 PMC3427019

[B170] Timmann C. Thye T. Vens M. Evans J. May J. Ehmen C. (2012). Genome-wide association study indicates two novel resistance loci for severe malaria. *Nature* 489 443–446. 10.1038/nature11334 22895189

[B171] Tuanyok A. Auerbach R. K. Brettin T. S. Bruce D. C. Munk A. C. Detter J. C. (2007). A horizontal gene transfer event defines two distinct groups within *Burkholderia pseudomallei* that have dissimilar geographic distributions. *J. Bacteriol.* 189 9044–9049. 10.1128/jb.01264-07 17933898 PMC2168593

[B172] Tuanyok A. Leadem B. R. Auerbach R. K. Beckstrom-Sternberg S. M. Beckstrom-Sternberg J. S. Mayo M. (2008). Genomic islands from five strains of *Burkholderia pseudomallei*. *BMC Genom.* 9:566. 10.1186/1471-2164-9-566 19038032 PMC2612704

[B173] Tuanyok A. Stone J. K. Mayo M. Kaestli M. Gruendike J. Georgia S. (2012). The Genetic and molecular basis of o-antigenic diversity in *Burkholderia pseudomallei* Lipopolysaccharide. *PLoS Neg. Trop. Dis.* 6:e1453. 10.1371/journal.pntd.0001453 22235357 PMC3250505

[B174] U’Ren J. M. Schupp J. M. Pearson T. Hornstra H. Friedman C. L. C. Smith K. L. (2007). Tandem repeat regions within the *Burkholderia pseudomallei* genome and their application for high resolution genotyping. *BMC Microbiol.* 7:23. 10.1186/1471-2180-7-23 17397553 PMC1853098

[B175] van den Broek T. Borghans J. A. M. van Wijk F. (2018). The full spectrum of human naive T cells. *Nat. Rev. Immunol.* 18 363–373. 10.1038/s41577-018-0001-y 29520044

[B176] Vander Broek C. W. Stevens J. M. (2017). Type III secretion in the melioidosis pathogen *Burkholderia pseudomallei*. *Front. Cell. Infect. Microbiol.* 7:255. 10.3389/fcimb.2017.00255 28664152 PMC5471309

[B177] Viberg L. T. Price E. P. Kidd T. J. Bell S. C. Currie B. J. Sarovich D. S. (2015). Whole-Genome Sequences of five *Burkholderia pseudomallei* isolates from Australian cystic fibrosis patients. *Genome Announc.* 3:e00254-15. 10.1128/genomeA.00254-15 PMC4400425 25883282

[B178] Viberg L. T. Sarovich D. S. Kidd T. J. Geake J. B. Bell S. C. Currie B. J. (2017). Within-host evolution of *Burkholderia pseudomallei* during Chronic Infection of Seven Australasian cystic fibrosis patients. *mBio* 8:e00356-17. 10.1128/mBio.00356-17 PMC5388805 28400528

[B179] Webb J. R. Rachlin A. Rigas V. Sarovich D. S. Price E. P. Kaestli M. (2019). Tracing the environmental footprint of the *Burkholderia pseudomallei* lipopolysaccharide genotypes in the tropical “Top End” of the Northern Territory, Australia. *PLoS Neg. Trop. Dis.* 13:e0007369. 10.1371/journal.pntd.0007369 31348781 PMC6701815

[B180] Weehuizen T. A. Prior J. L. van der Vaart T. W. Ngugi S. A. Nepogodiev S. A. Field R. A. (2015). Differential toll-like receptor-signalling of *Burkholderia pseudomallei* lipopolysaccharide in murine and human models. *PLoS One* 10:e0145397. 10.1371/journal.pone.0145397 26689559 PMC4687033

[B181] West T. E. Chantratita N. Chierakul W. Limmathurotsakul D. Wuthiekanun V. Myers N. D. (2013). Impaired TLR5 functionality is associated with survival in melioidosis. *J. Immunol.* 190 3373–3379. 10.4049/jimmunol.1202974 23447684 PMC3607401

[B182] West T. E. Chierakul W. Chantratita N. Limmathurotsakul D. Wuthiekanun V. Emond M. J. (2012). Toll-like receptor 4 region genetic variants are associated with susceptibility to melioidosis. *Genes Immun.* 13 38–46. 10.1038/gene.2011.49 21776015 PMC3483087

[B183] West T. E. Ernst R. K. Jansson-Hutson M. J. Skerrett S. J. (2008). Activation of Toll-like receptors by *Burkholderia pseudomallei*. *BMC Immunol.* 9:46. 10.1186/1471-2172-9-46 18691413 PMC2527550

[B184] Whitlock G. C. Deeraksa A. Qazi O. Judy B. M. Taylor K. Propst K. L. (2010). Protective response to subunit vaccination against intranasal *Burkholderia mallei* and *B. pseudomallei* challenge. *Proc. Vaccinol.* 2 73–77. 10.1016/j.provac.2010.03.013 24379895 PMC3874274

[B185] Wiersinga W. J. Wieland C. W. Dessing M. C. Chantratita N. Cheng A. C. Limmathurotsakul D. (2007). Toll-like receptor 2 impairs host defense in gram-negative sepsis caused by *Burkholderia pseudomallei* (Melioidosis). *PLoS Med.* 4:e248. 10.1371/journal.pmed.0040248 17676990 PMC1950213

[B186] Wikraiphat C. Charoensap J. Utaisincharoen P. Wongratanacheewin S. Taweechaisupapong S. Woods D. E. (2009). Comparative in vivo and in vitro analyses of putative virulence factors of *Burkholderia pseudomallei* using lipopolysaccharide, capsule and flagellin mutants. *FEMS Immunol. Med. Microbiol.* 56 253–259. 10.1111/j.1574-695x.2009.00574.x 19549172

[B187] Willcocks S. J. Denman C. C. Atkins H. S. Wren B. W. (2016). Intracellular replication of the well-armed pathogen *Burkholderia pseudomallei*. *Curr. Opin. Microbiol.* 29 94–103. 10.1016/j.mib.2015.11.007 26803404

[B188] Williams N. L. Morris J. L. Rush C. M. Ketheesan N. (2015). Plasmacytoid dendritic cell bactericidal activity against *Burkholderia pseudomallei*. *Microb. Infect.* 17 311–316. 10.1016/j.micinf.2014.12.007 25532693

[B189] Williams R. C. Muller Y. L. Hanson R. L. Knowler W. C. Mason C. C. Bian L. (2011). HLA-DRB1 reduces the risk of type 2 diabetes mellitus by increased insulin secretion. *Diabetologia* 54 1684–1692. 10.1007/s00125-011-2122-8 21484216 PMC6432927

[B190] Yang J. Lee S. H. Goddard M. E. Visscher P. M. (2011). GCTA: a tool for genome-wide complex trait analysis. *Am. J. Hum. Genet.* 88 76–82. 10.1016/j.ajhg.2010.11.011 21167468 PMC3014363

[B191] Yu Y. Kim H. S. Chua H. H. Lin C. H. Sim S. H. Lin D. (2006). Genomic patterns of pathogen evolution revealed by comparison of *Burkholderia pseudomallei*, the causative agent of melioidosis, to avirulent *Burkholderia thailandensis*. *BMC Microbiol.* 6:46. 10.1186/1471-2180-6-46 16725056 PMC1508146

[B192] Zhang X. Zhuchenko O. Kuspa A. Soldati T. (2016). Social amoebae trap and kill bacteria by casting DNA nets. *Nat. Commun.* 7:10938. 10.1038/ncomms10938 PMC4773522 26927887

[B193] Zhao W. Rasheed A. Tikkanen E. Lee J.-J. Butterworth A. S. Howson J. M. M. (2017). Identification of new susceptibility loci for type 2 diabetes and shared etiological pathways with coronary heart disease. *Nat. Genet.* 49 1450–1457. 28869590 10.1038/ng.3943 PMC5844224

[B194] Zou N. Tsai S. Feng S. H. Newsome T. Kim H. Y. Li B. (2008). Relationship between antigenicity and pathogenicity for *Burkholderia pseudomallei* and *Burkholderia mallei* revealed by a large panel of mouse MAbs. *Hybridoma (Larchmt)* 27 231–240. 18707541 10.1089/hyb.2008.0012

[B195] Zou S. Li J. Zhou H. Frech C. Jiang X. Chu J. S. (2014). Mutational landscape of intrahepatic cholangiocarcinoma. *Nat. Commun.* 5:5696. 10.1038/ncomms6696 25526346

