# Corrigendum: An Evolutionary Arms Race Between *Burkholderia pseudomallei* and Host Immune System: What Do We Know?

**DOI:** 10.3389/fmicb.2021.801975

**Published:** 2021-11-22

**Authors:** Chalita Chomkatekaew, Phumrapee Boonklang, Apiwat Sangphukieo, Claire Chewapreecha

**Affiliations:** ^1^Mahidol-Oxford Tropical Medicine Research Unit (MORU), Bangkok, Thailand; ^2^Bioinformatics and Systems Biology Program, School of Bioresource and Technology, King Mongkut's University of Technology Thonburi, Bangkok, Thailand; ^3^Wellcome Sanger Institute, Hinxton, United Kingdom

**Keywords:** melioidosis, evolution, burkholderia, host immune system, genetic variants

In the original article, there was a mistake in Figure 1 as published. The annotation of core and accessory genes in the Figure 1 was misplaced. The corrected figure appears below.

**Figure 1:**
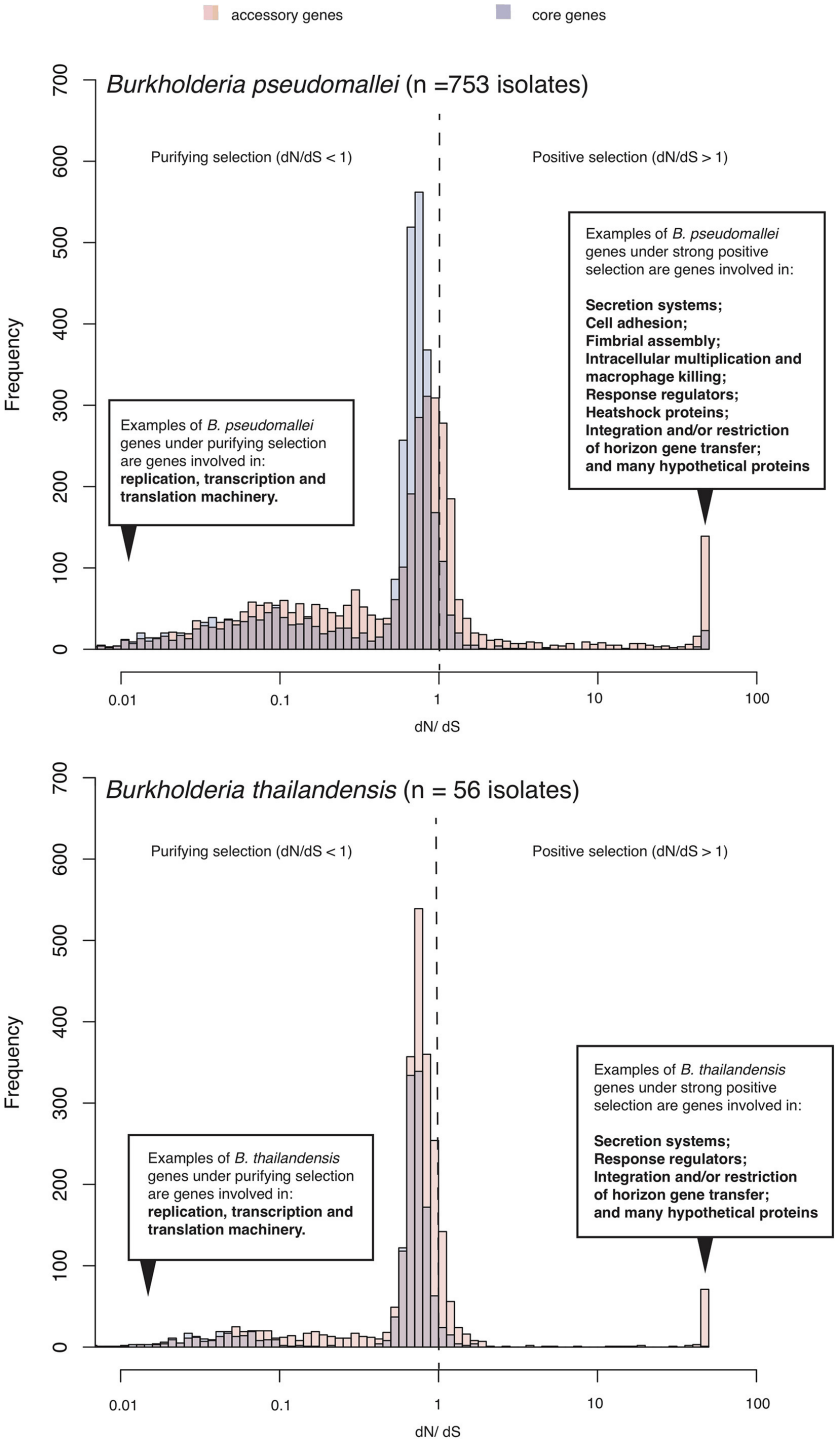
Selection pressure acting on *B. pseudomallei* population. The histogram summarizes ranges of *dN/dS* calculated from predicted coding sequences from a collection of diverse *B. pseudomallei* population from northeast Thailand (Chewapreecha et al., 2019), and *B. thailandensis* genomes from the public database. *B. pseudomallei* and *B. thailandensis* have highly plastic genomes comprising of at least two chromosomes of ~7–8 Mb in size when combined. Using a pan-genome approach, all coding sequences could be categorized as “core” (present in all genomes) or “accessory” (variably present across studied genomes). Accessory genes display an elevated level of *dN/dS* which is signatures of positive selection or more relaxed purifying selection.

The authors apologize for this error and state that this does not change the scientific conclusions of the article in any way. The original article has been updated.

## Publisher's Note

All claims expressed in this article are solely those of the authors and do not necessarily represent those of their affiliated organizations, or those of the publisher, the editors and the reviewers. Any product that may be evaluated in this article, or claim that may be made by its manufacturer, is not guaranteed or endorsed by the publisher.

